# Supplementary material for: The negative adipogenesis regulator Dlk1 is transcriptionally regulated by Ifrd1 (TIS7) and translationally by its orthologue Ifrd2 (SKMc15)
Source: eLife. 2023 Aug 21;12:e88350. doi: 10.7554/eLife.88350 (PMC10468205; doi:10.7554/eLife.88350)
Supplement: Figure 1—figure supplement 1—source data 1. [file elife-88350-fig1-figsupp1-data1.pdf]

Energy Metabolism

Jan Rozman
Ann-Elisabeth Schwarz
Anna Dewert, Brigitte Herrmann
Martin Klingenspor *Technische Universität München*

General information

We conducted a 21 hours indirect calorimetry trial monitoring gas exchange (oxygen consumption and carbon dioxide production), activity (distance and rearing), and food intake.

We measured body mass before and after the trial and determined rectal body temperature (around 10 am before transfer of mice back to their home cages). Body composition was determined by q NMR (MiniSpec).

In a first step for statistical analysis genotype effects were tested using 1 way ANOVA.

Food intake and energy expenditure were analysed using a linear model including body mass as a co-variate. There same approach was used to analyse shifts in body composition.

Indirect calorimetry – results overview

Parameter	Male		ANOVA (genotype)<br>*LM (body mass as co-variate)	
	Control	Mutant	Genotype	body mass
	n= 6	n= 7		
	mean ± sd	mean ± sd	p-value	p-value
**Body mass (g**	31.1 ± 2.6	20.4 ± 1.9	< 0.0001	n/a
**Body temperature (°C)**	36.56 ± 0.6	36.71 ± 0.5	0.6515	n/a
***Food intake (g)**	4.0 ± 0.4	3.4 ± 0.5	0.5869	0.1277
***Mean VO_2 (ml h^{-1})**	107.28 ± 8.41	81.12 ± 5.69	0.6441	0.0241
***Min VO_2 (ml h^{-1})**	78.83 ± 8.26	57.71 ± 8.86	0.9047	0.1219
***Max VO_2 (ml h^{-1})**	144.33 ± 6.98	107.29 ± 7.59	0.1944	0.0082
**Mean RER**	0.89 ± 0.01	0.90 ± 0.02	0.3754	n/a
**Mean DistD (cm 20 min^{-1})**	928 ± 173	874 ± 219	0.6399	n/a
**Mean Z (rearing 20min^{-1})**	116 ± 34	96 ± 25	0.2444	n/a

Figures – general results

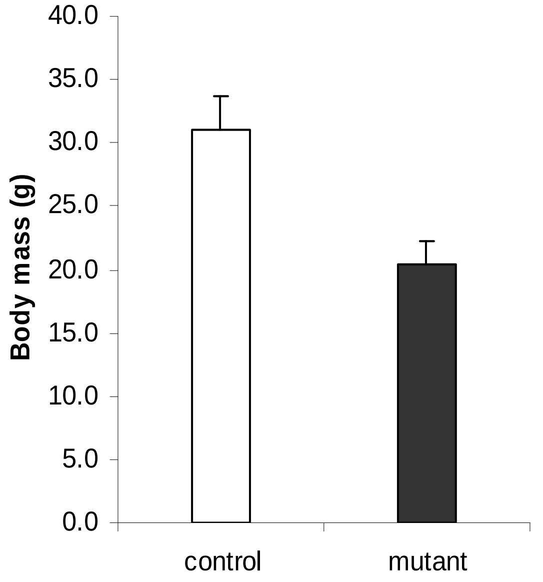

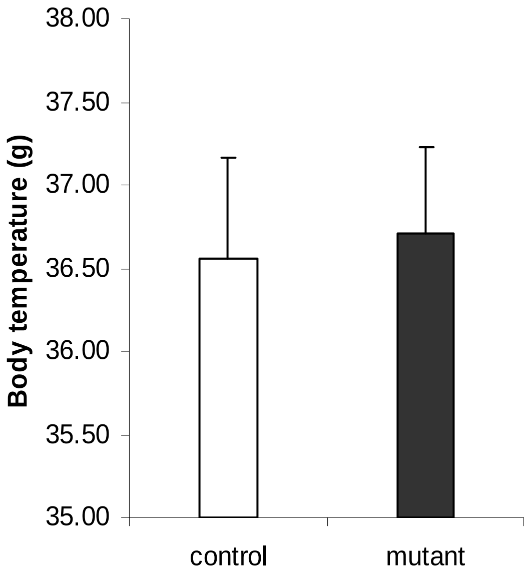

Body composition (qNMR)*

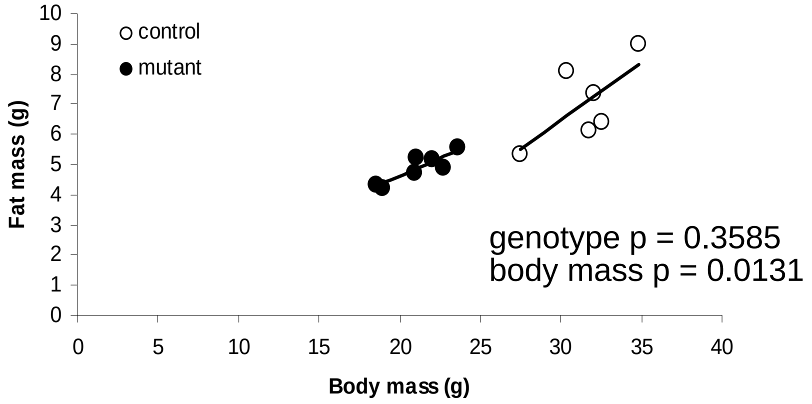

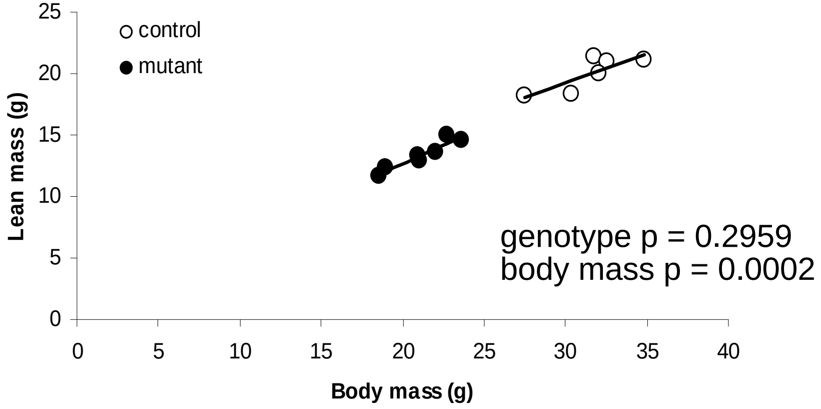

*statistics: linear regression model including body mass as co-variate

Indirect Calorimetry: VO2 over time

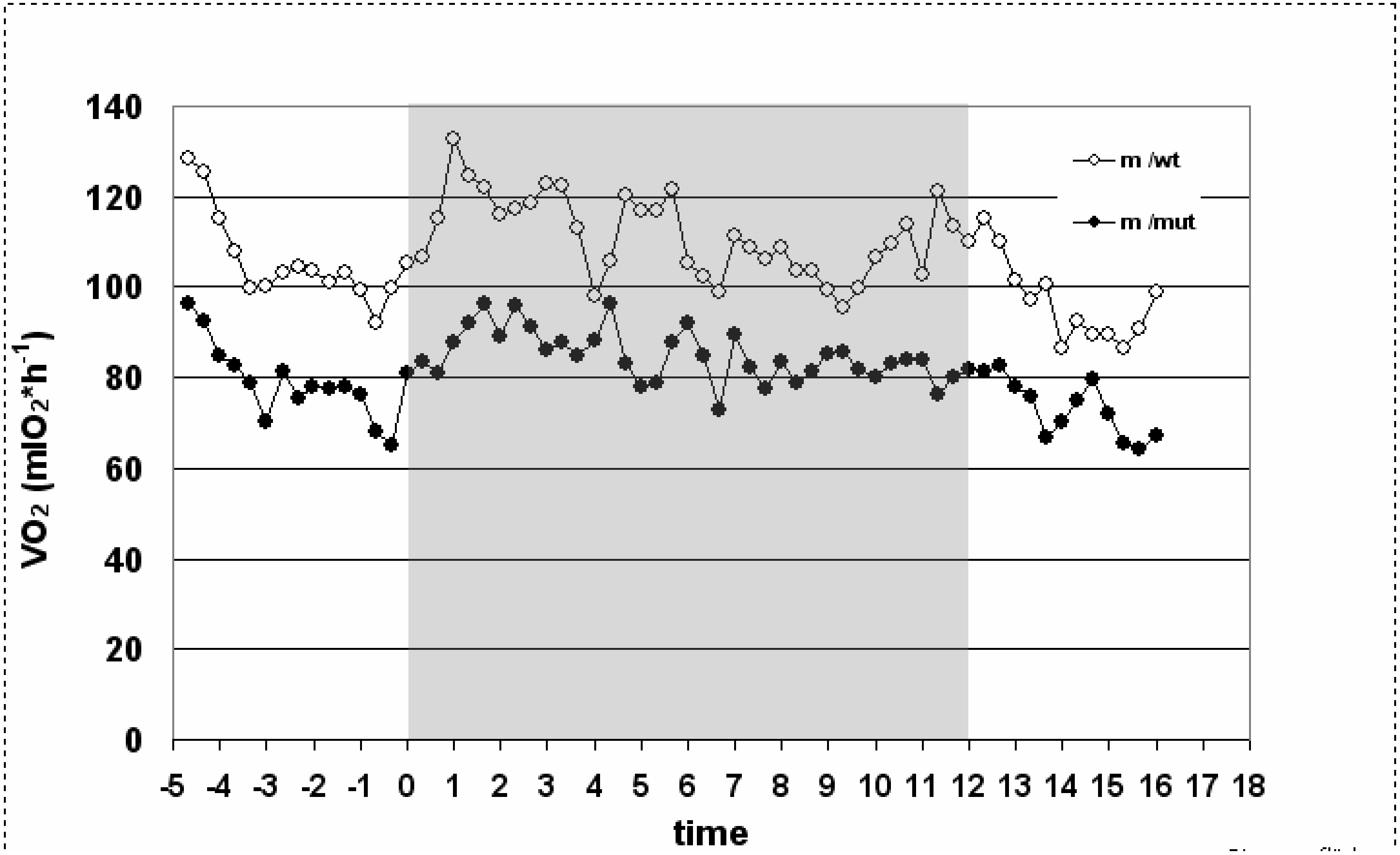

Energy expenditure and food consumption

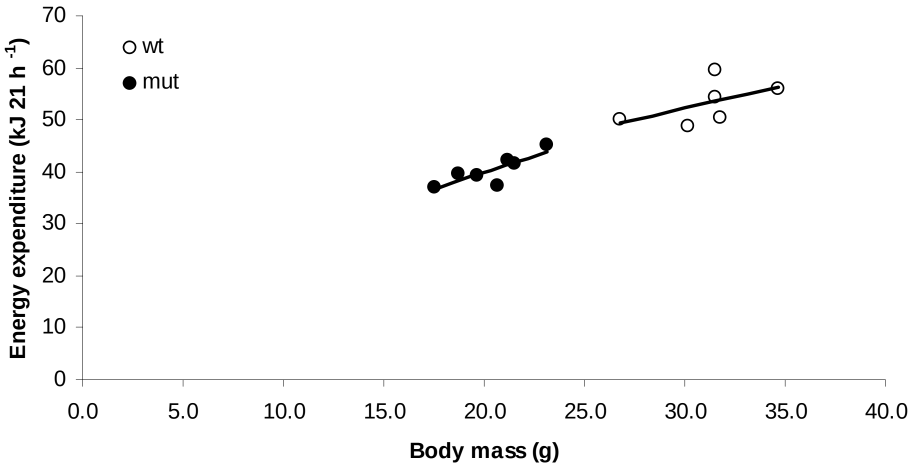

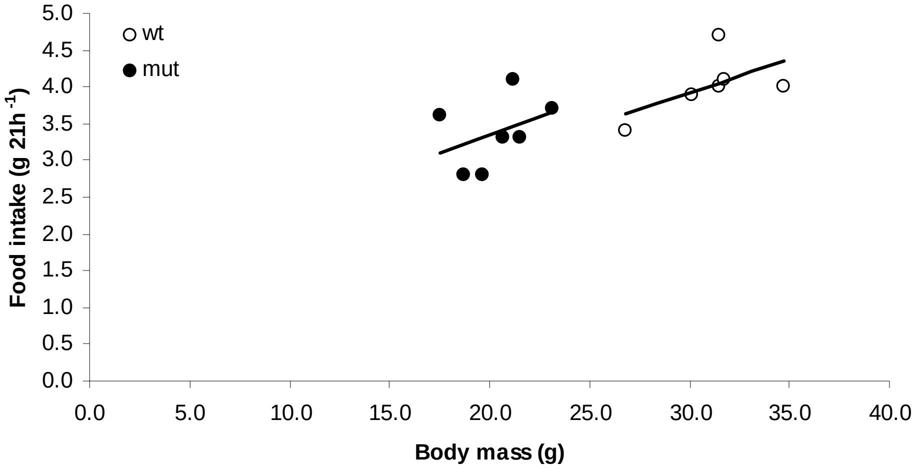

RER: substrate utilization

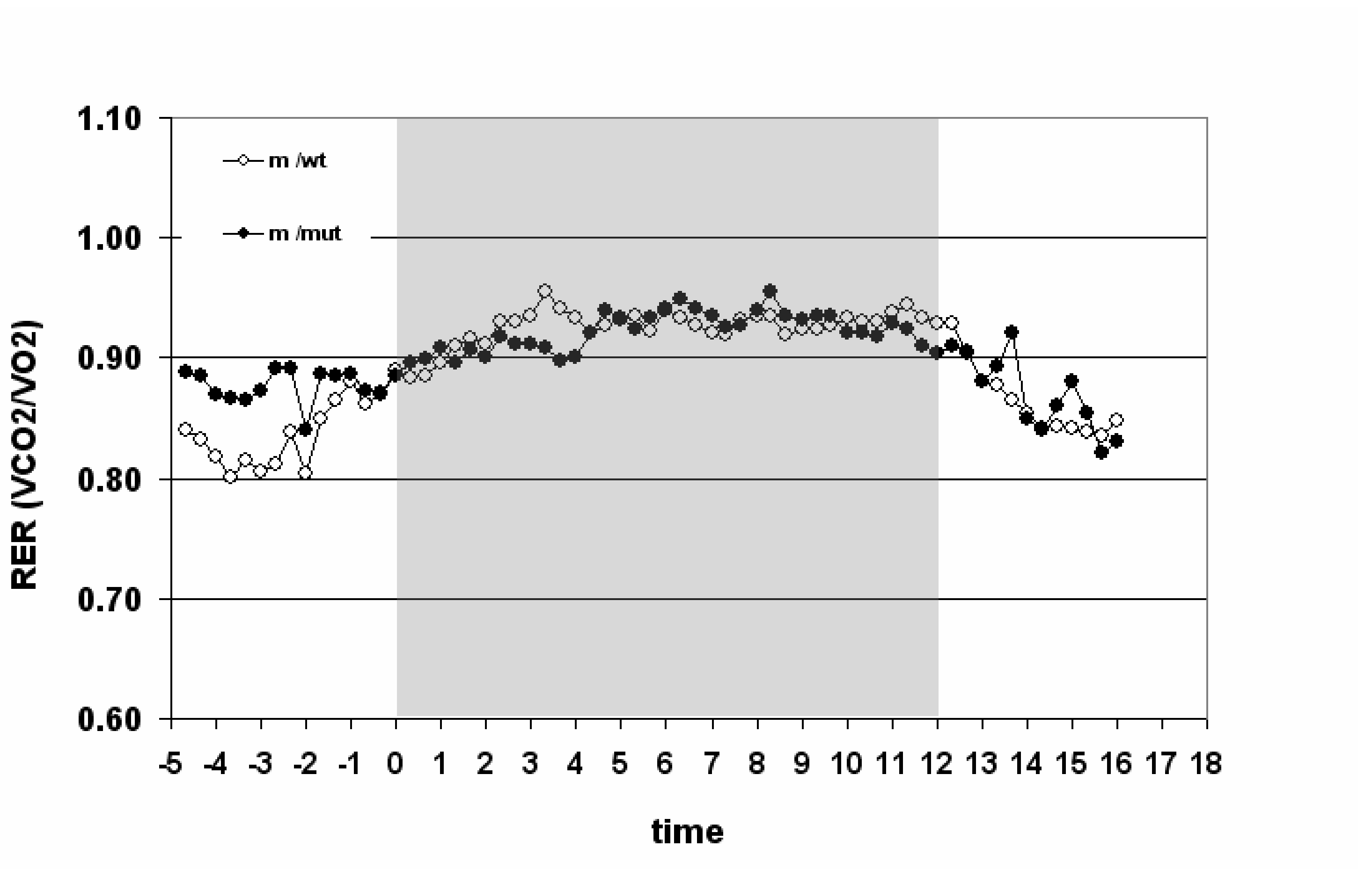

Locomotor activity and rearing

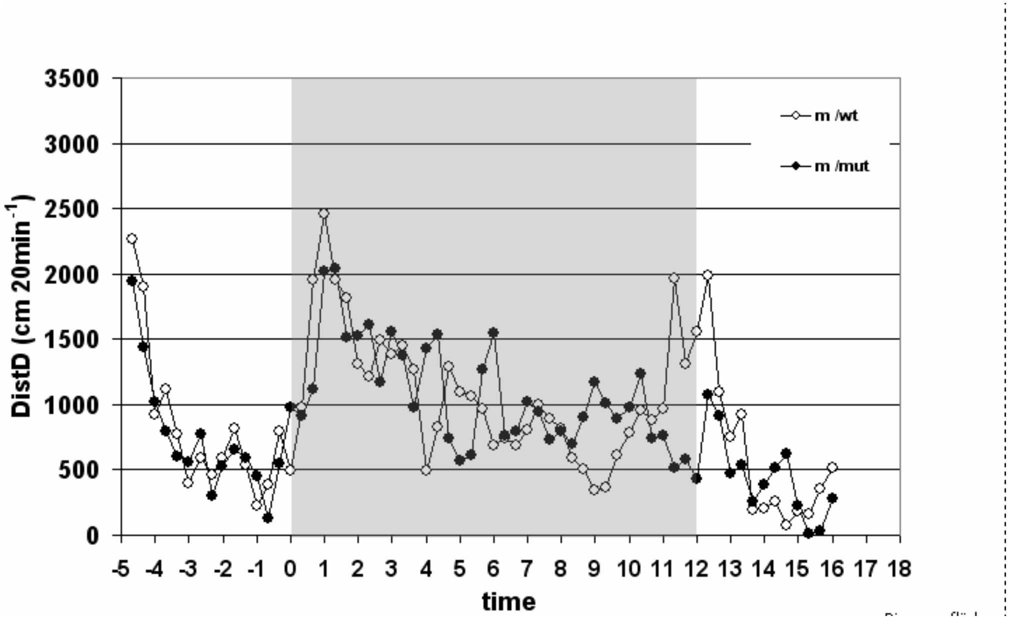

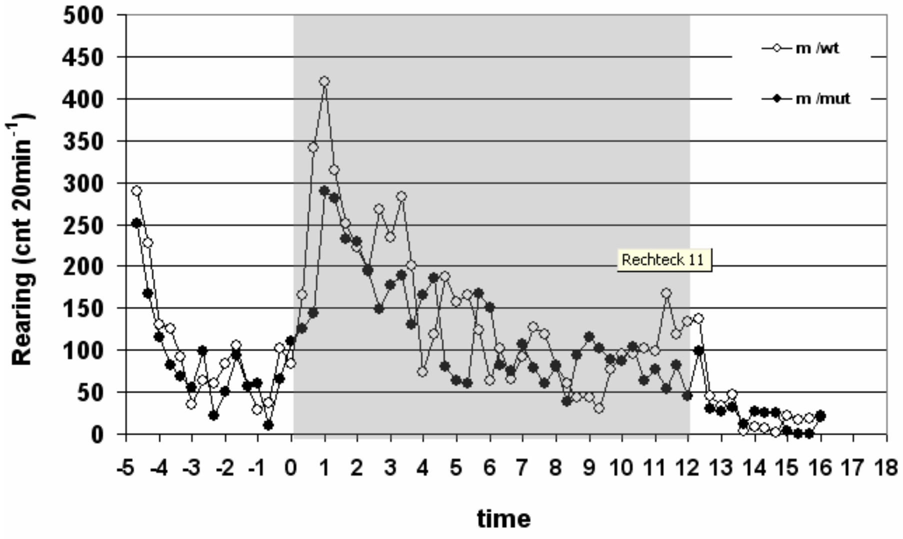

Summary

The reduction in body mass between control and mutant mice was confirmed.

Rectal body temperature determined after the indirect calorimetry trial did not differ.

Both in control and mutant mice we observed the typical daily pattern in oxygen consumption during the IC trial. Mean oxygen consumption over 21 hours was correlated with body mass. Absolute VO2 was lower in mutants, however, adjusting for the body mass difference showed that VO2 was more or less as expected for reduced body mass. Using this approach no genotype effect on oxygen consumption could be detected.

There was also no clear genotype effect on food intake that seemed to be slightly increased in mutant mice.

Apart from minor differences observed during the first hours of the trial, RER was exactly the same in control and mutant mice. There was no indication for a shift in substrate utilization.

No effects could be detected in distance travelled and rearing behaviour.

It could be interesting to feed the mice a high fat diet and monitor energy balance as well as shifts in substrate utilization. Further details need to be discussed.
