# Supplementary figures and images for: The negative adipogenesis regulator Dlk1 is transcriptionally regulated by Ifrd1 (TIS7) and translationally by its orthologue Ifrd2 (SKMc15)
Source: eLife. 2023 Aug 21;12:e88350. doi: 10.7554/eLife.88350 (PMC10468205; doi:10.7554/eLife.88350)

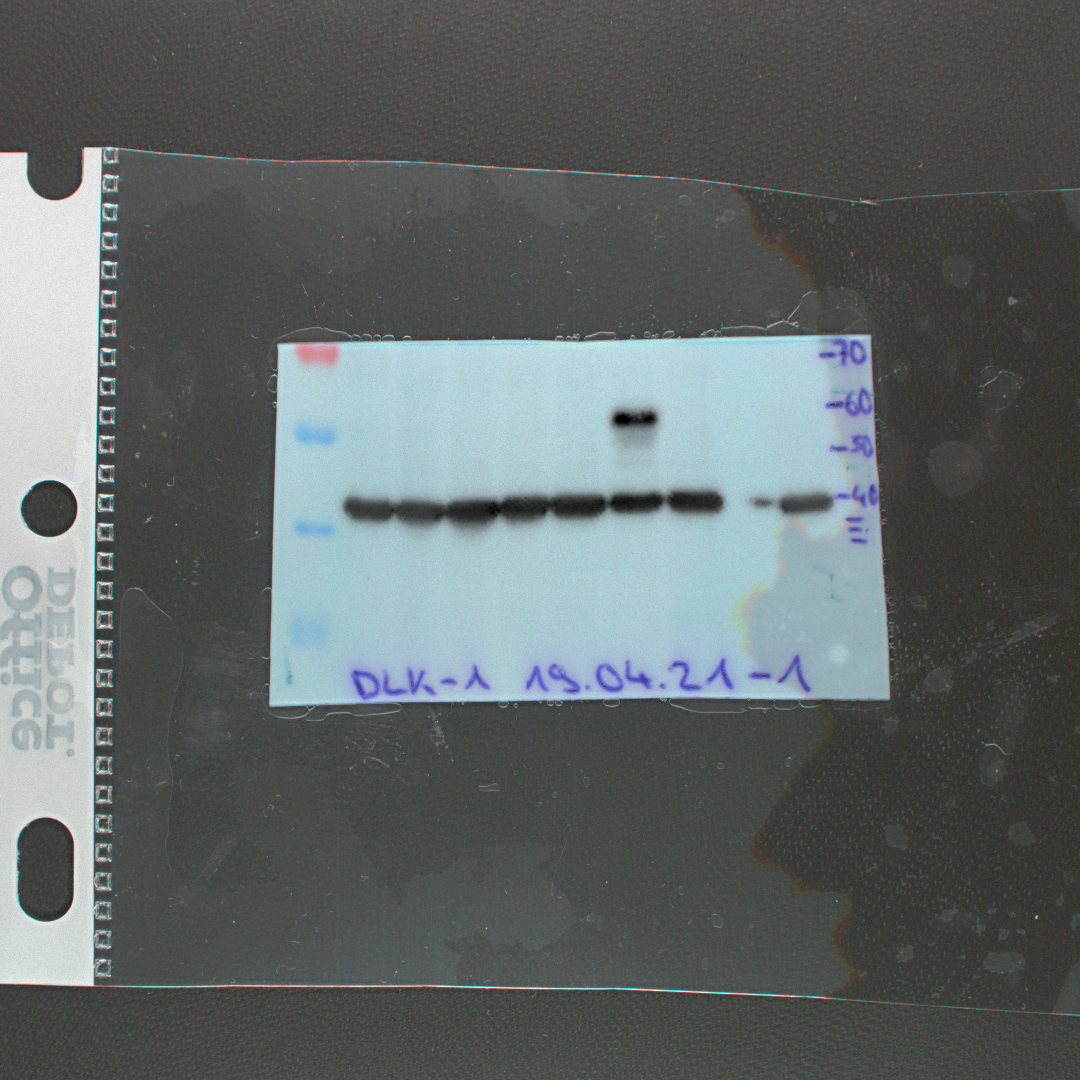

Supplement: Figure 1—figure supplement 2—source data 1. [file elife-88350-fig1-figsupp2-data1.zip › Figure 1 - figure supplement 2/figure 1 - figure supplement 2I/b_actin.Tif]

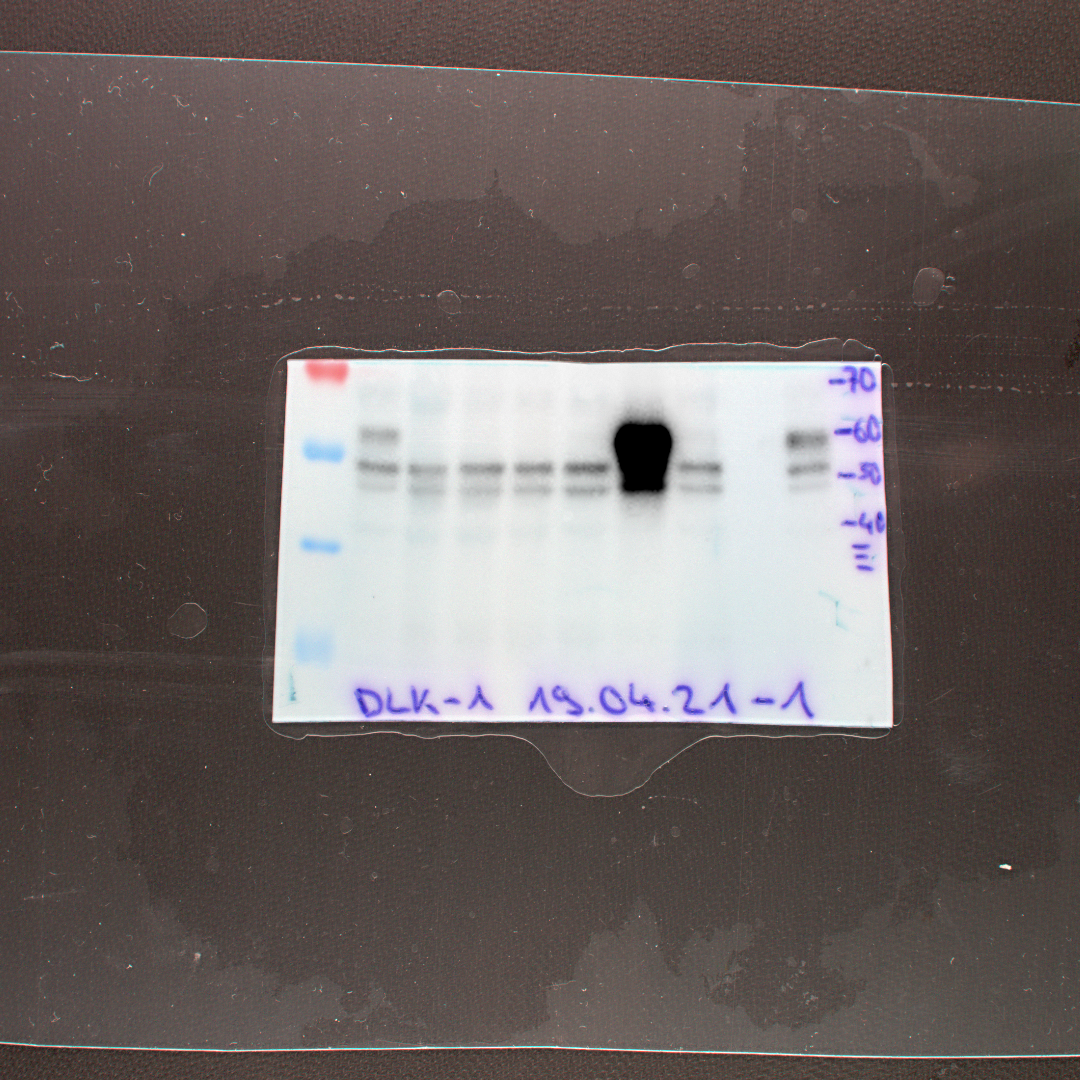

Supplement: Figure 1—figure supplement 2—source data 1. [file elife-88350-fig1-figsupp2-data1.zip › Figure 1 - figure supplement 2/figure 1 - figure supplement 2I/IFRD1.Tif]

I

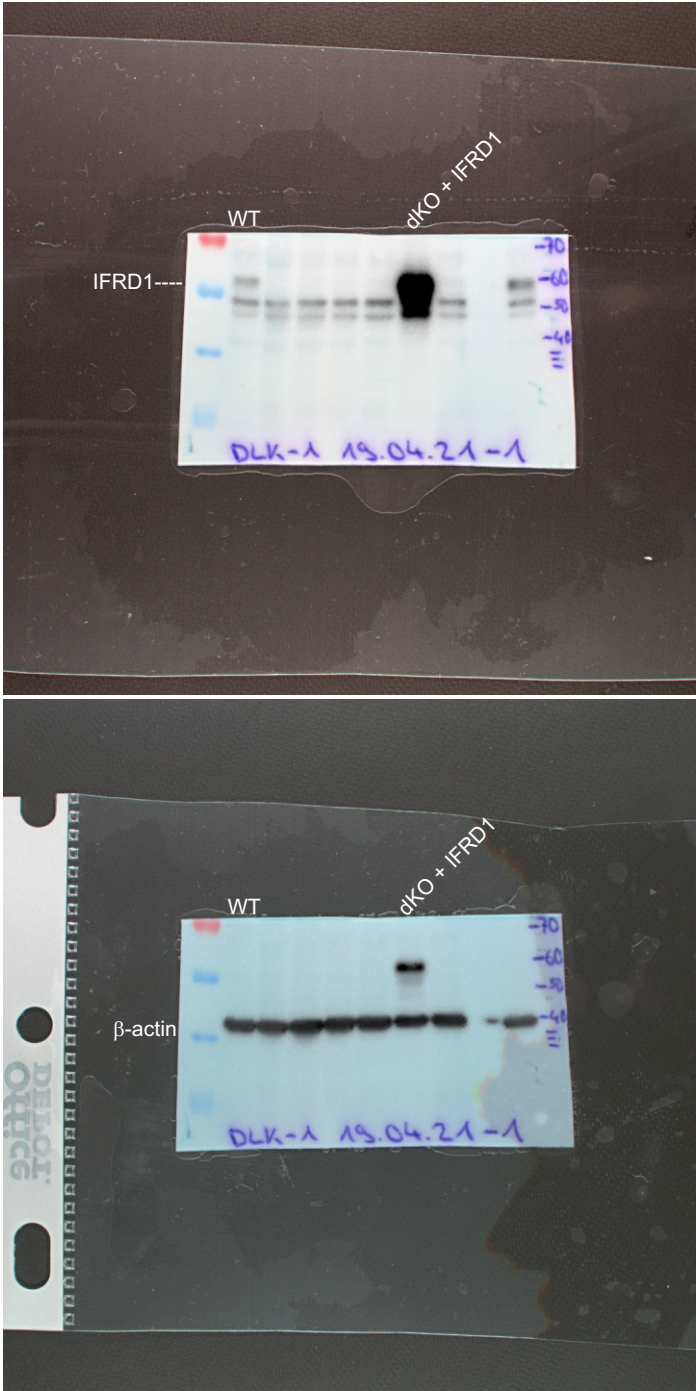

J

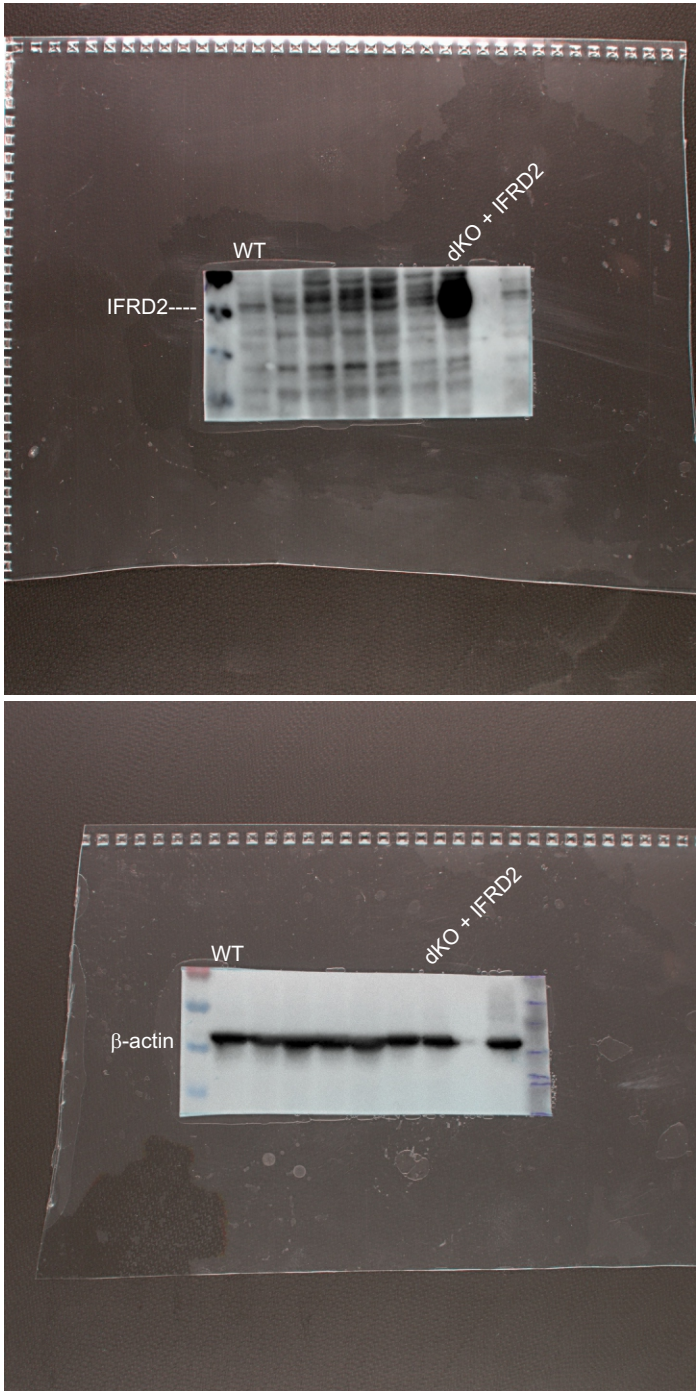

Supplement: Figure 1—figure supplement 2—source data 1. [file elife-88350-fig1-figsupp2-data1.zip › Figure 1 - figure supplement 2/figure 1 - figure supplement 2I/manuscript Figure 1 - supplement 2I,J labeled.pdf]

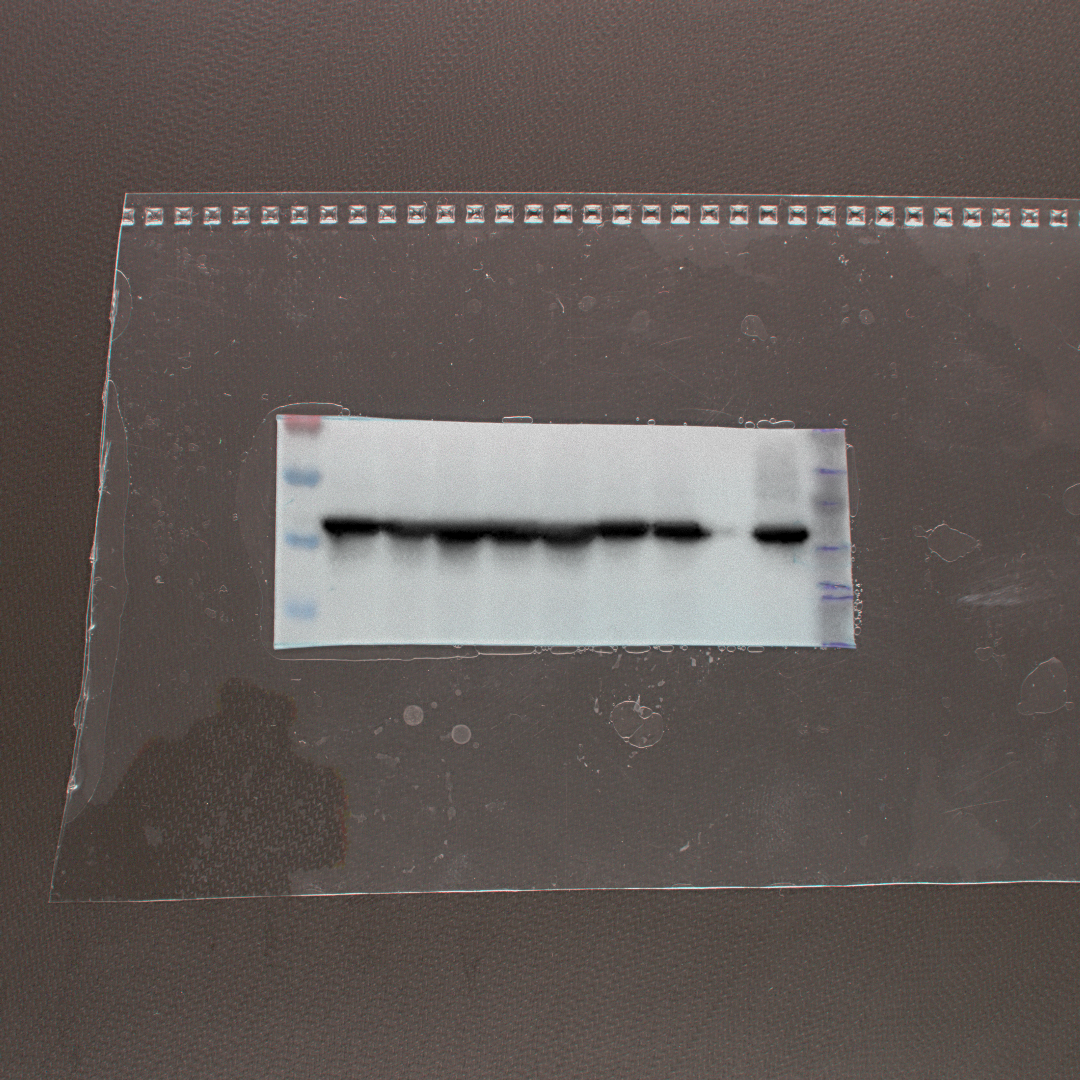

Supplement: Figure 1—figure supplement 2—source data 1. [file elife-88350-fig1-figsupp2-data1.zip › Figure 1 - figure supplement 2/figure 1 - figure supplement 2J/beta-actin.Tif]

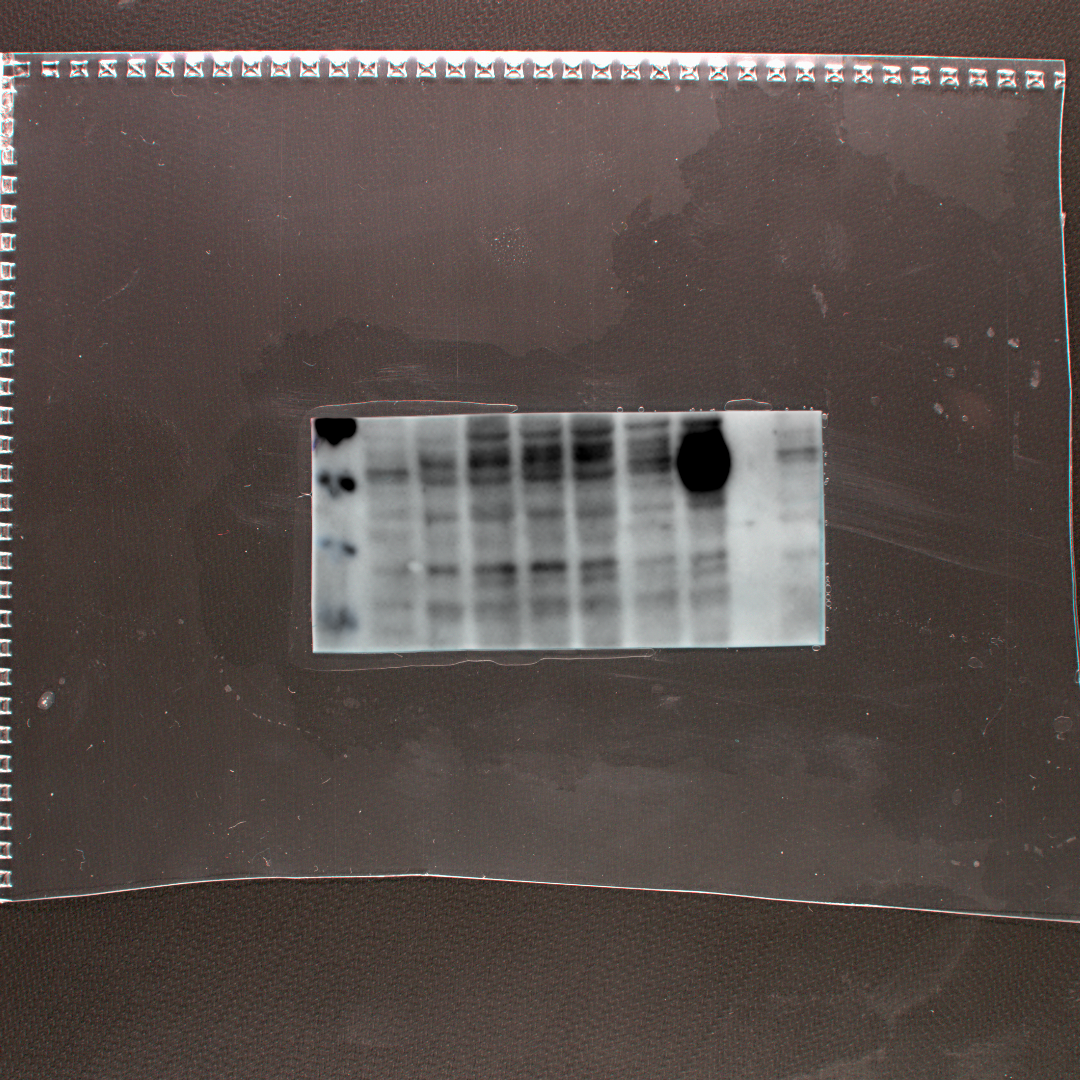

Supplement: Figure 1—figure supplement 2—source data 1. [file elife-88350-fig1-figsupp2-data1.zip › Figure 1 - figure supplement 2/figure 1 - figure supplement 2J/IFRD2.Tif]

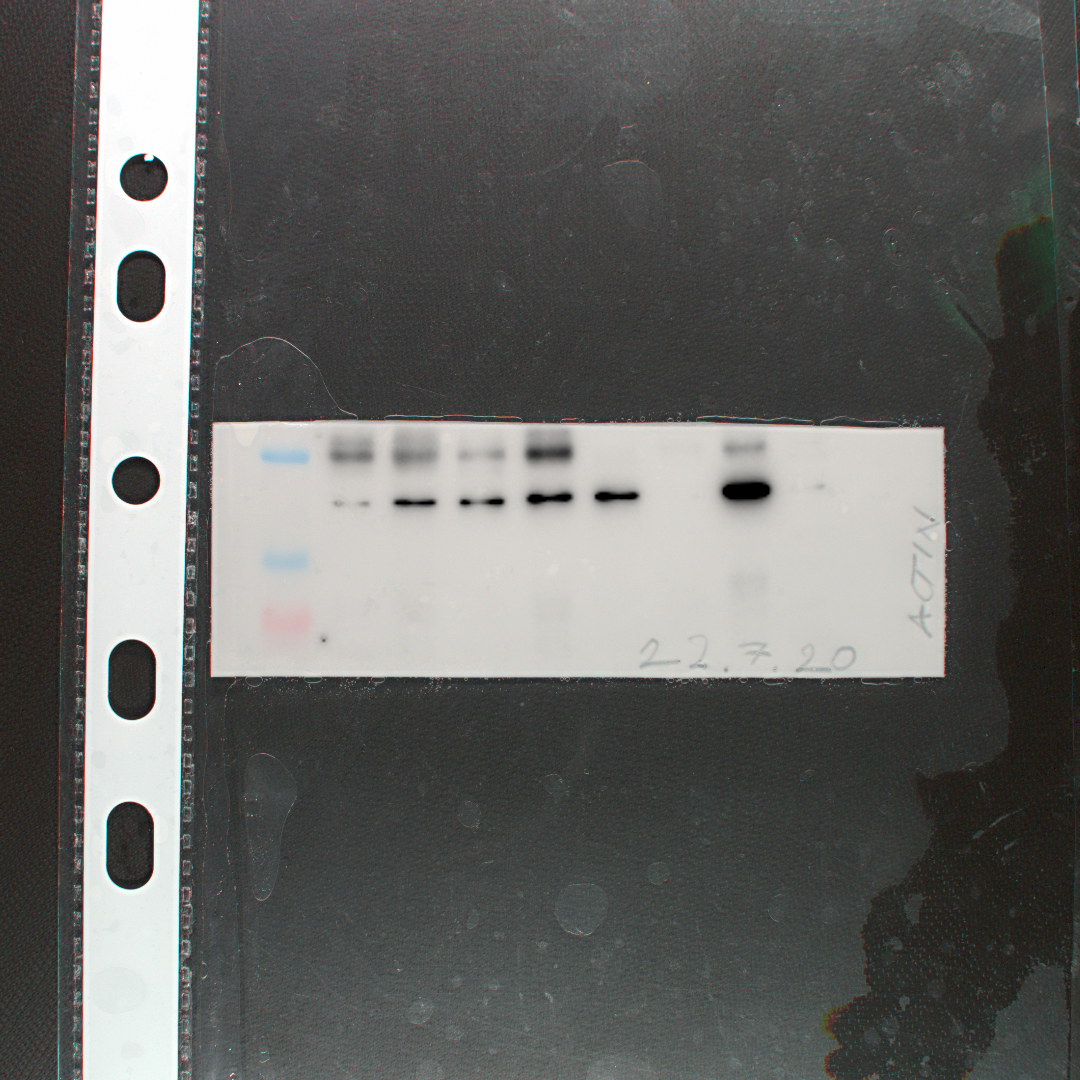

Supplement: Figure 2—source data 1. [file elife-88350-fig2-data1.zip › Figure 2D - source data/actin for bcatenin.Tif]

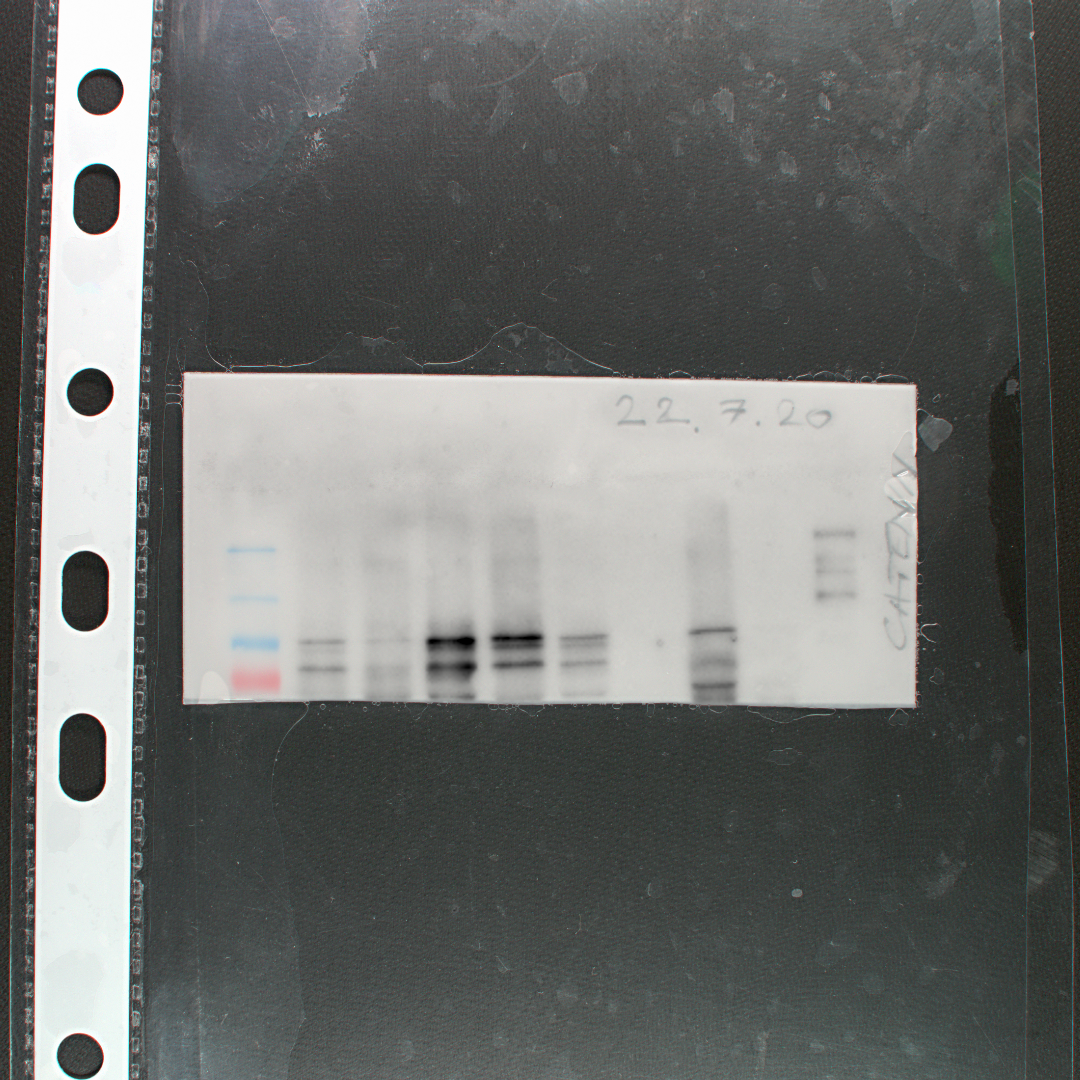

Supplement: Figure 2—source data 1. [file elife-88350-fig2-data1.zip › Figure 2D - source data/bcatenin.Tif]

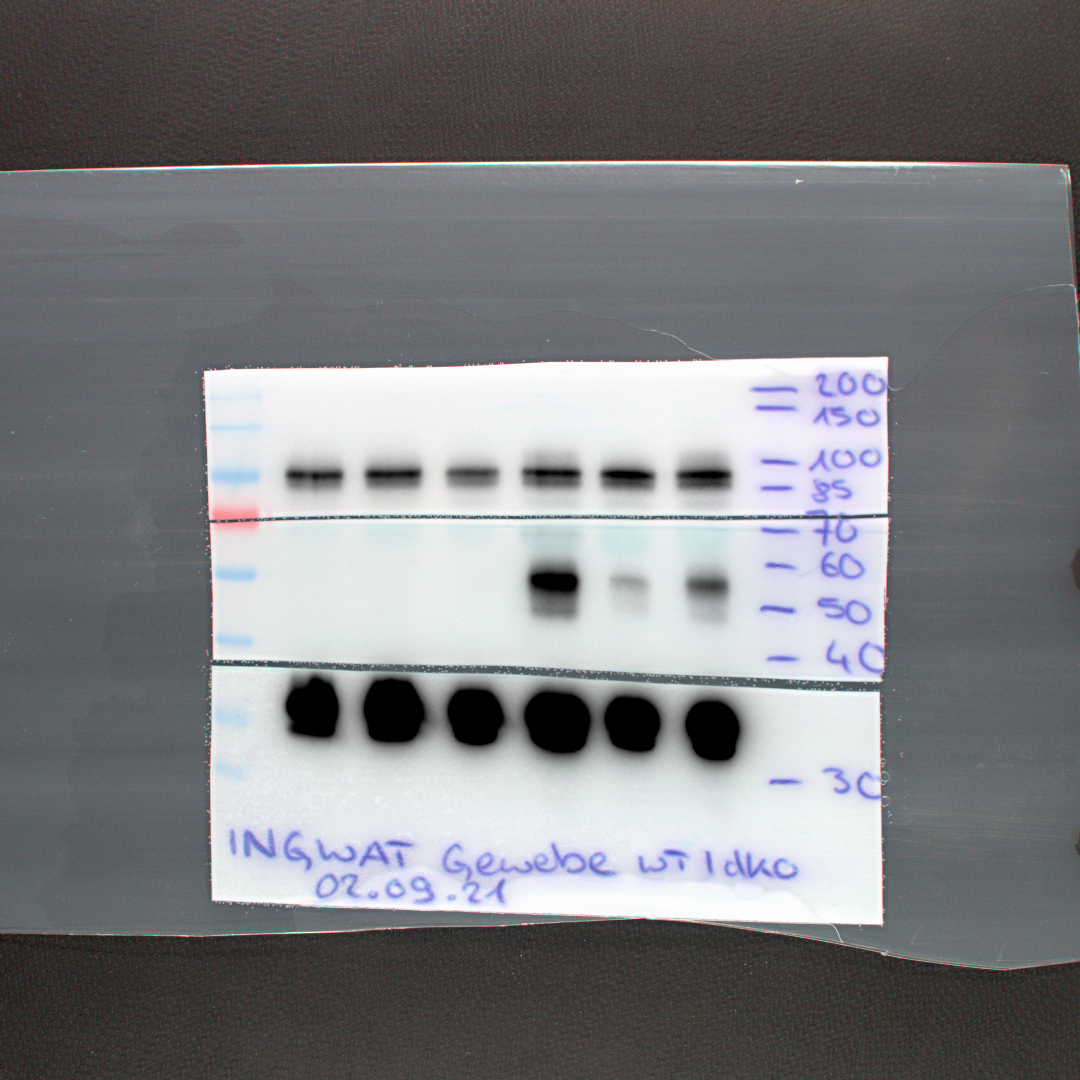

Supplement: Figure 2—source data 1. [file elife-88350-fig2-data1.zip › Figure 2D - source data/DLK1.Tif]

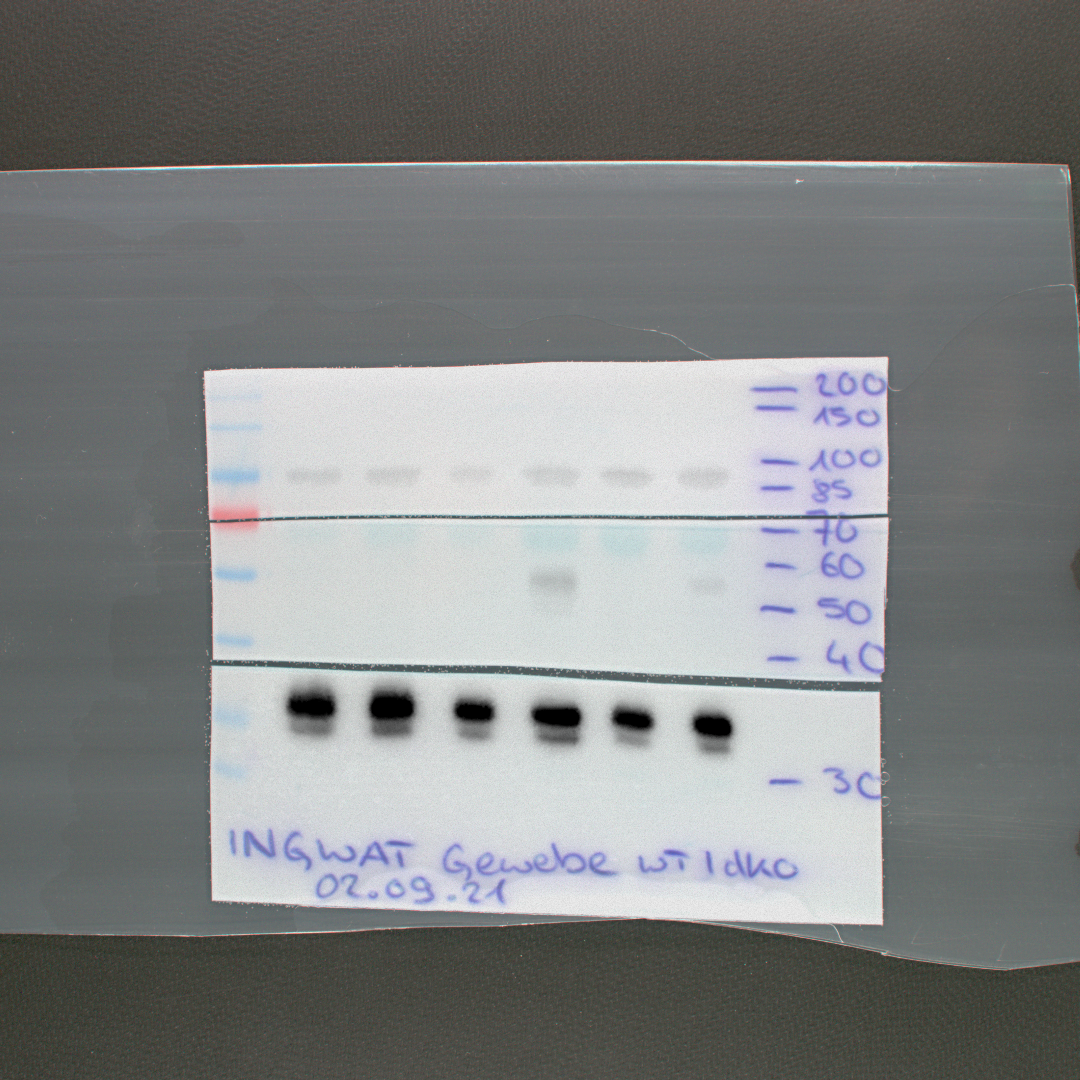

Supplement: Figure 2—source data 1. [file elife-88350-fig2-data1.zip › Figure 2D - source data/GAPDH for DLK1.Tif]

D

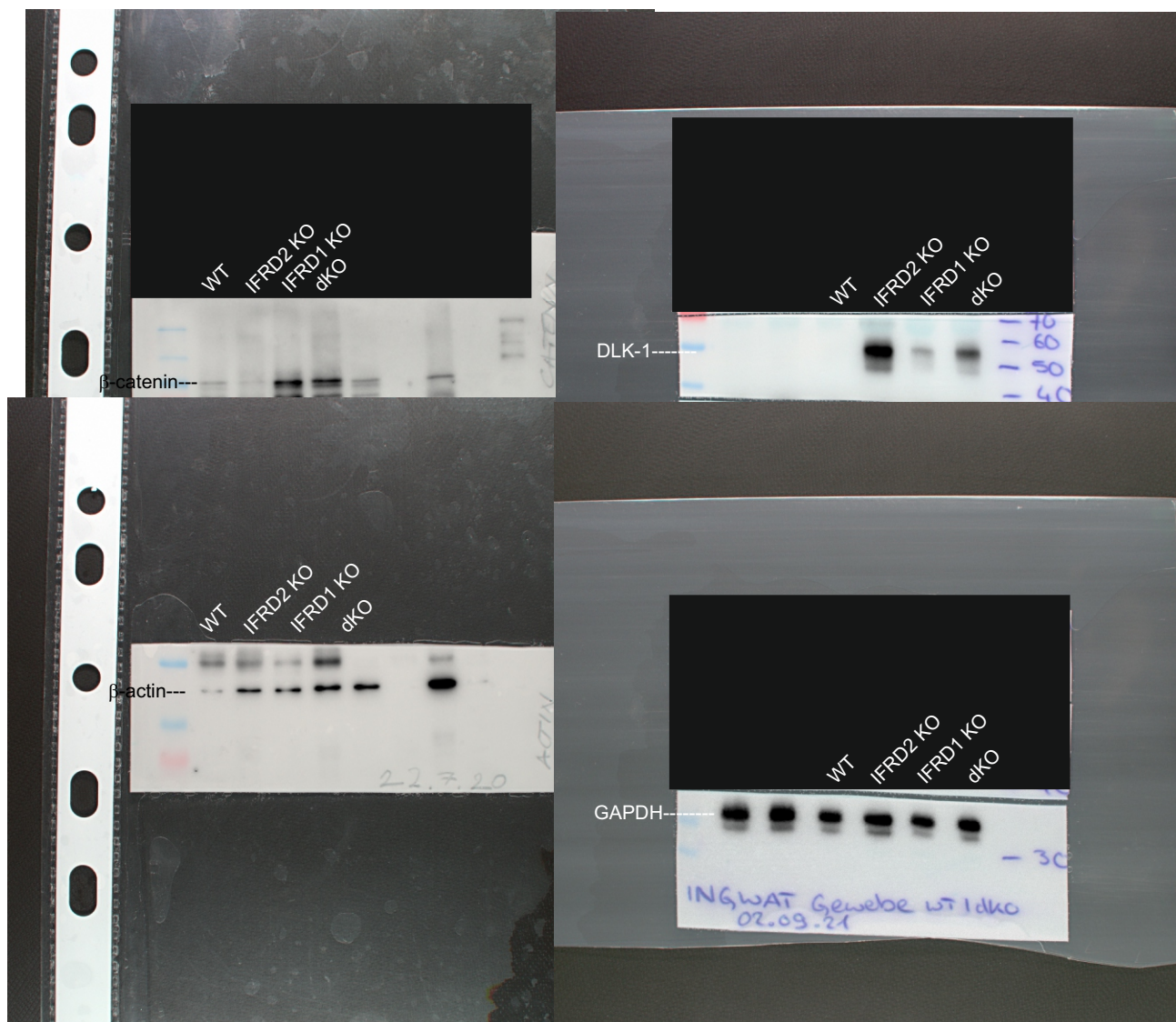

Supplement: Figure 2—source data 1. [file elife-88350-fig2-data1.zip › Figure 2D - source data/manuscript Fig 2D labeled.pdf]

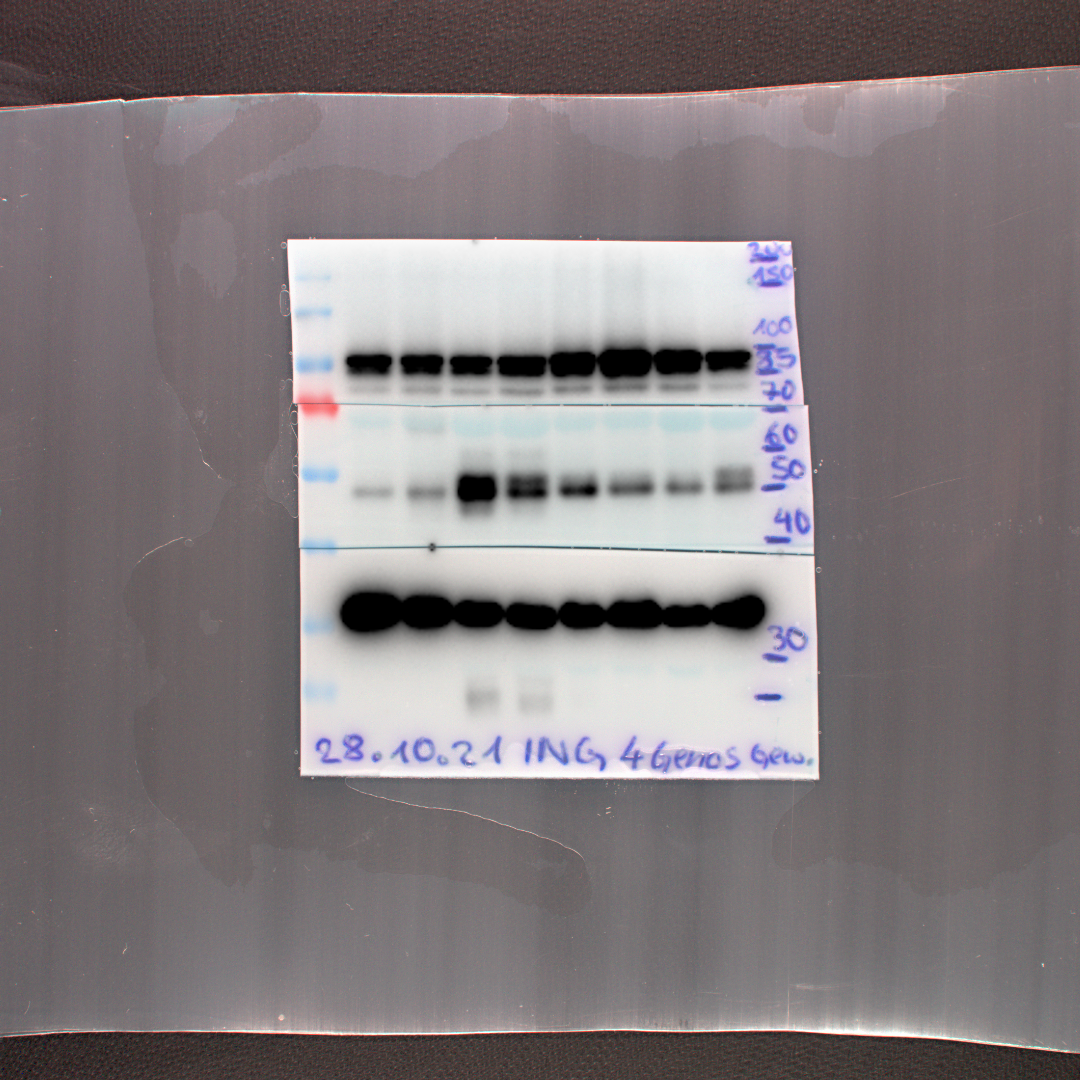

Supplement: Figure 2—source data 2. [file elife-88350-fig2-data2.zip › Figure 2E - source data/DLK-1 center lane 2 and 3.tif]

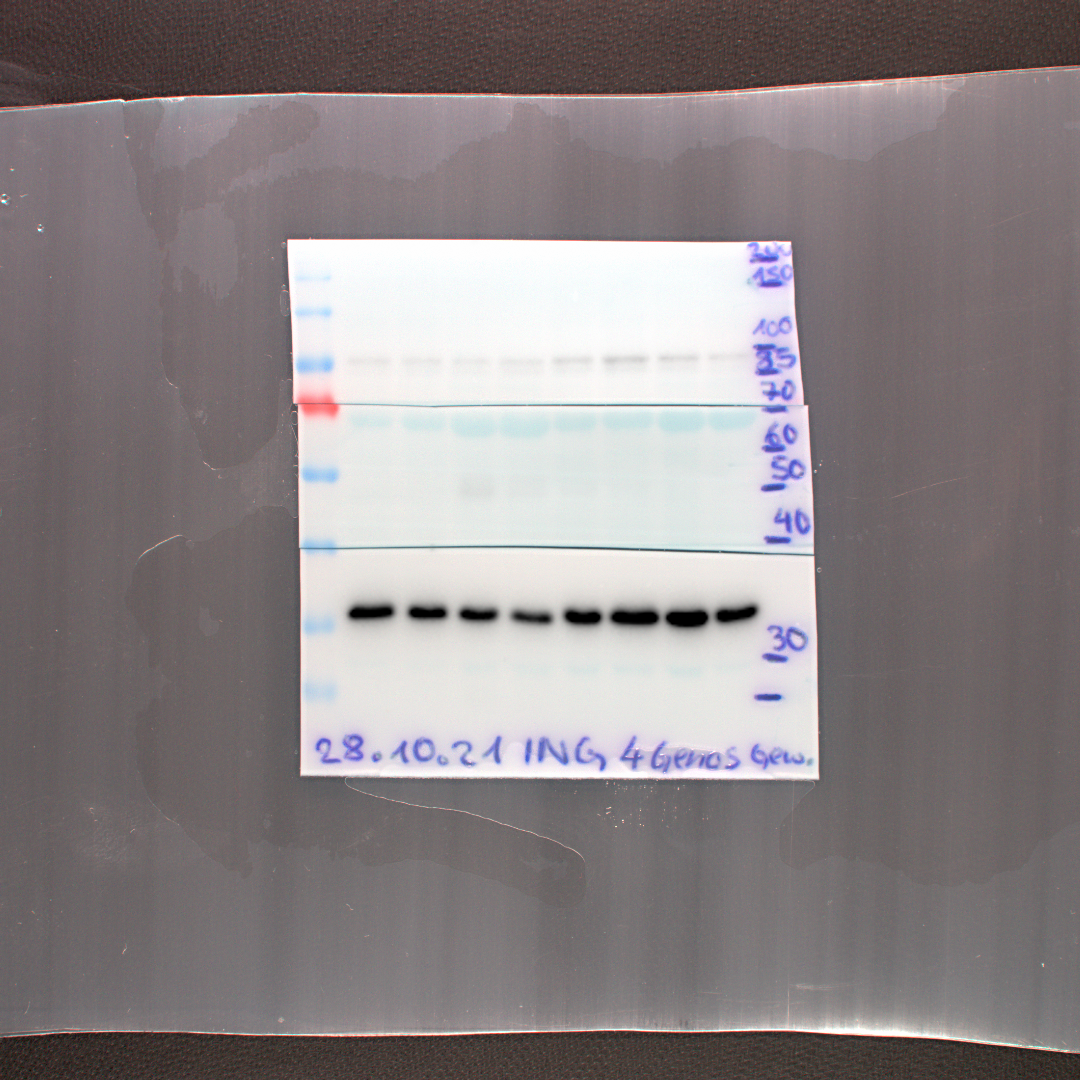

Supplement: Figure 2—source data 2. [file elife-88350-fig2-data2.zip › Figure 2E - source data/GAPDH bottom lane 2 and 3.tif]

E

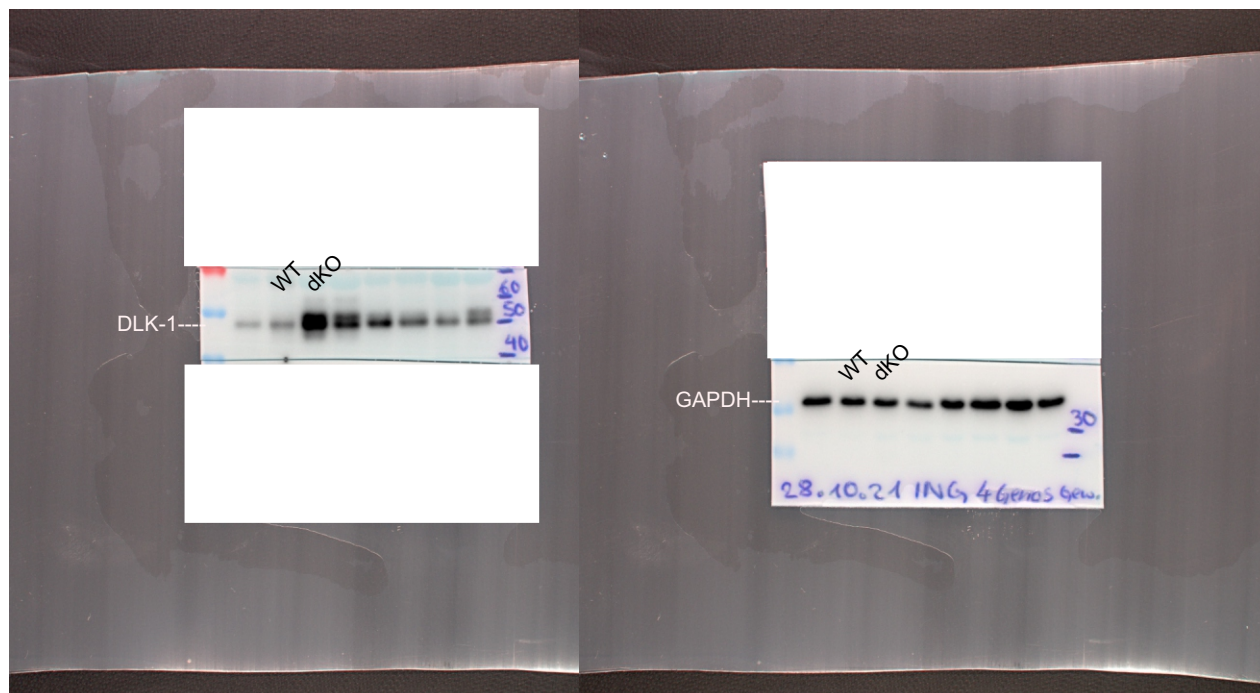

Supplement: Figure 2—source data 2. [file elife-88350-fig2-data2.zip › Figure 2E - source data/manuscript Fig 2E labeled.pdf]

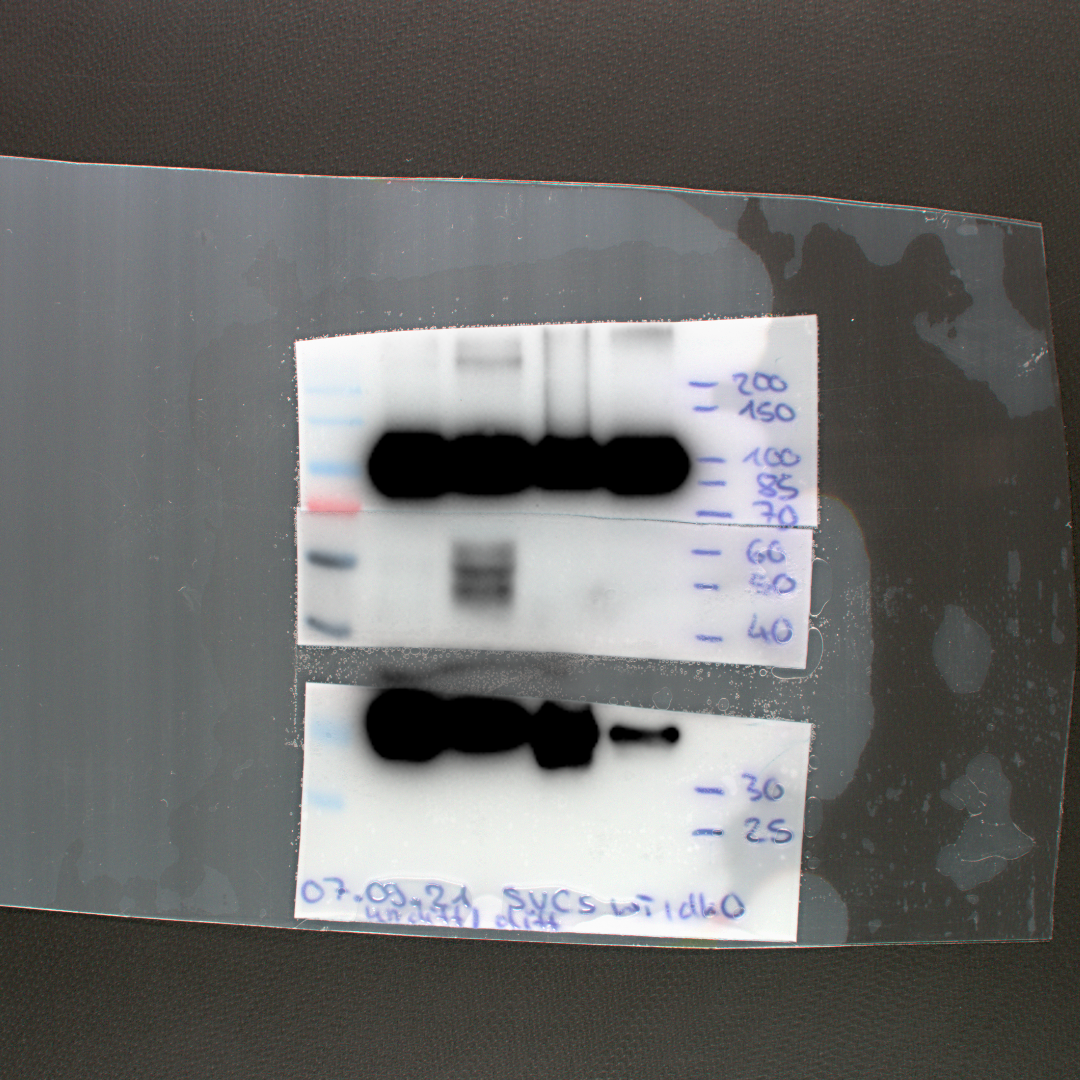

Supplement: Figure 2—source data 3. [file elife-88350-fig2-data3.zip › Figure 2G - source data/DLK-1_center membrane_lane 1 and 2.tif]

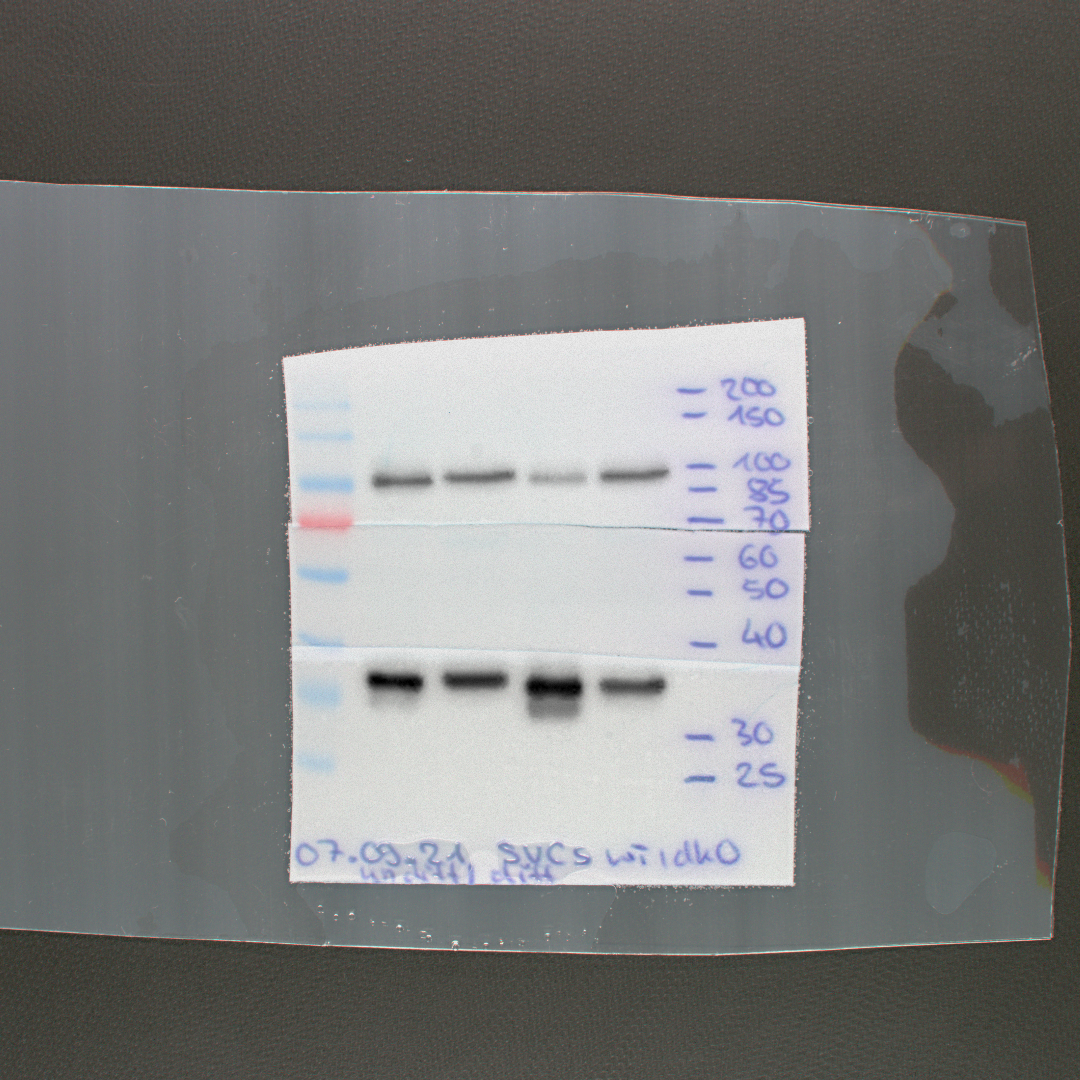

Supplement: Figure 2—source data 3. [file elife-88350-fig2-data3.zip › Figure 2G - source data/GAPDH_bottom membrane_lane 1 and 2.tif]

G

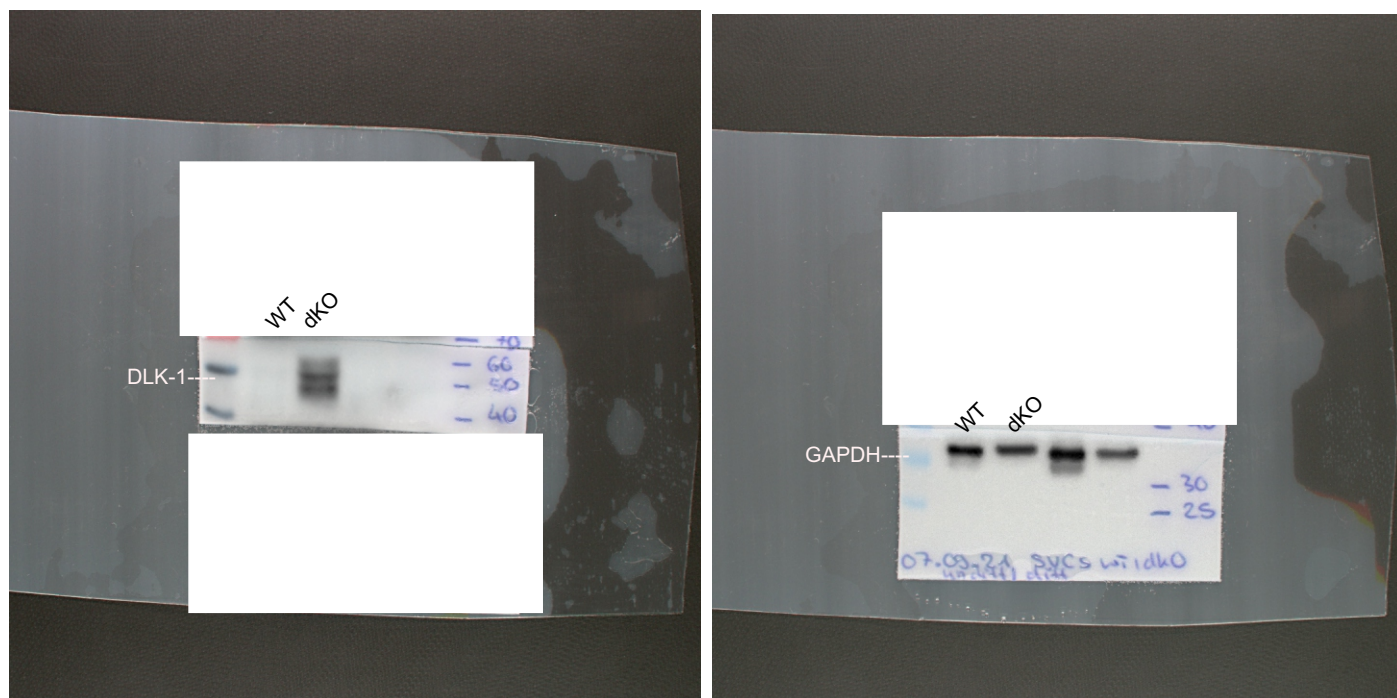

Supplement: Figure 2—source data 3. [file elife-88350-fig2-data3.zip › Figure 2G - source data/manuscript Fig 2G labeled.pdf]

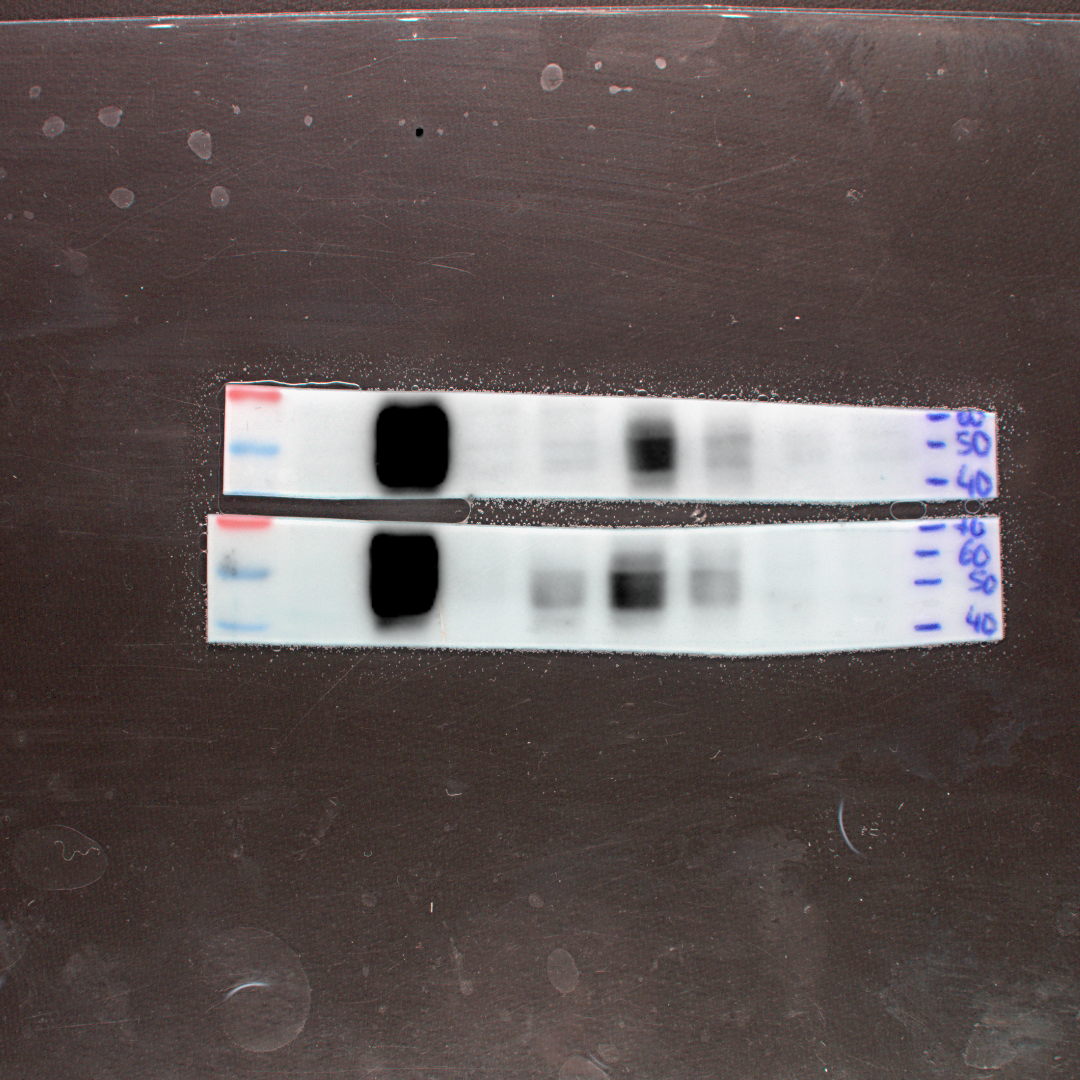

Supplement: Figure 3—source data 1. [file elife-88350-fig3-data1.zip › Figure 3B - source data/DLK1 lower membrane lanes 1-2 and 5-8.Tif]

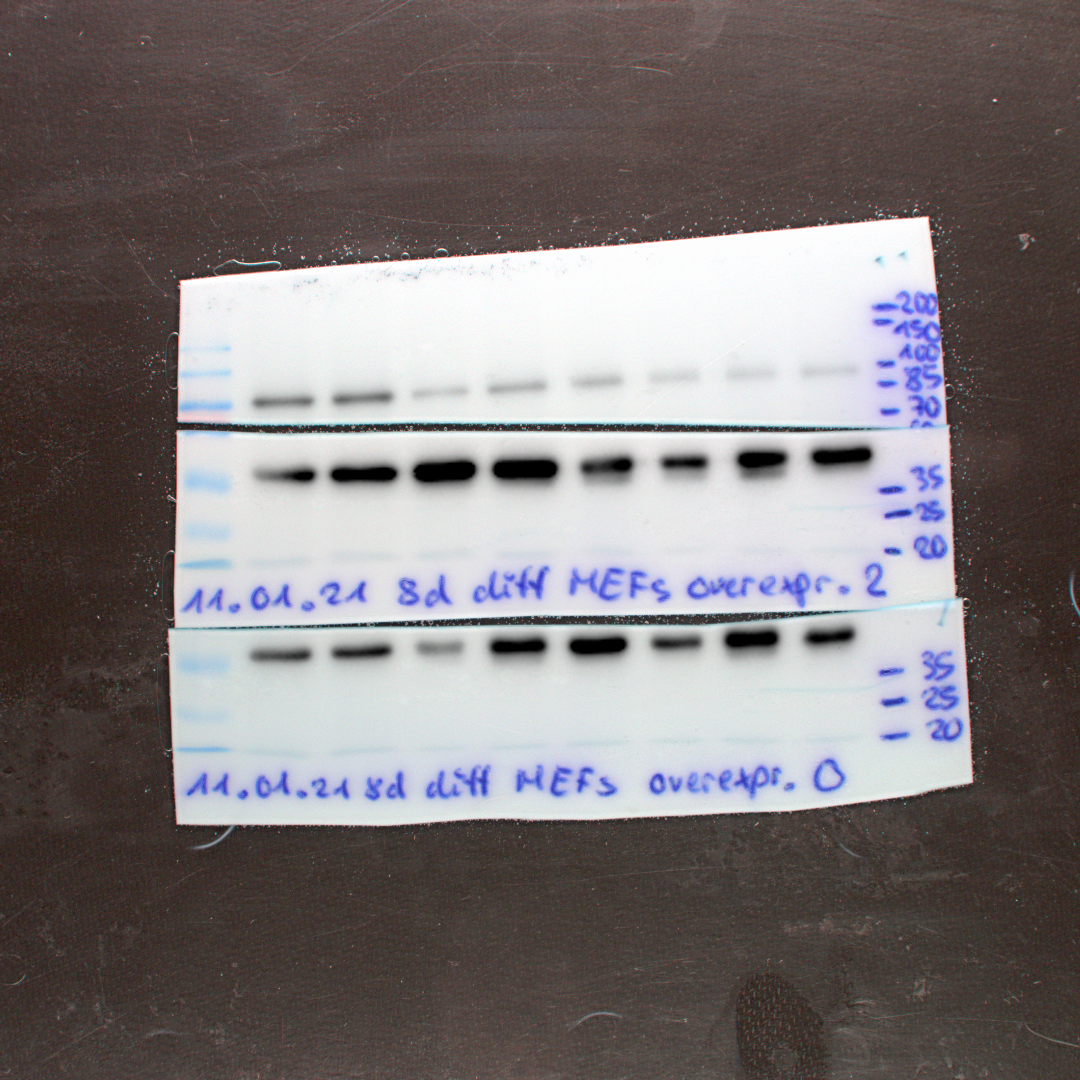

Supplement: Figure 3—source data 1. [file elife-88350-fig3-data1.zip › Figure 3B - source data/GAPDH lowest membrane lanes 1-2 and 5-8.Tif]

B

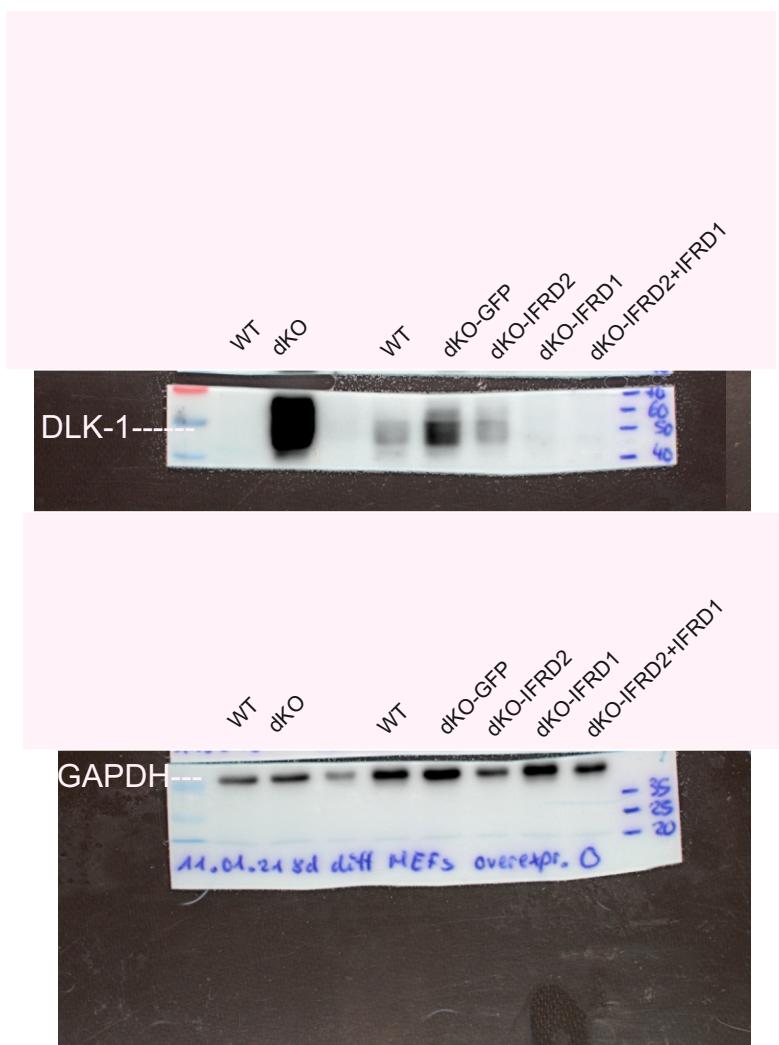

Supplement: Figure 3—source data 1. [file elife-88350-fig3-data1.zip › Figure 3B - source data/manuscript Fig 3B labeled.pdf]

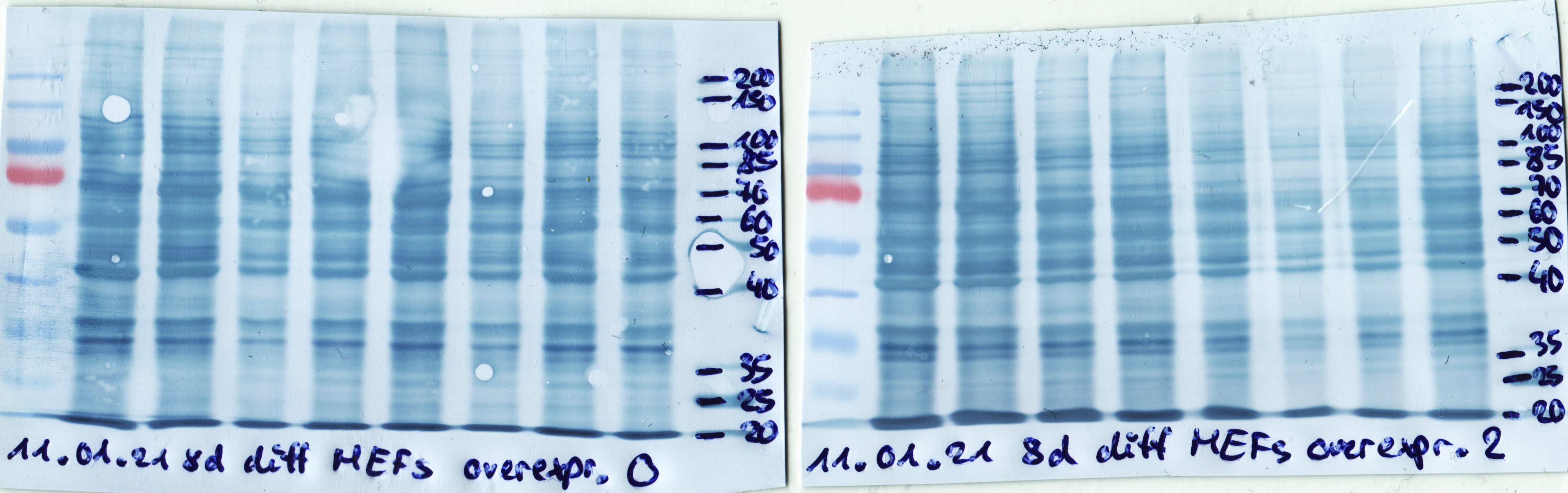

Supplement: Figure 3—figure supplement 1—source data 1. [file elife-88350-fig3-figsupp1-data1.zip › Figure 3 - figure supplement 1/amido black - right membrane.tif]

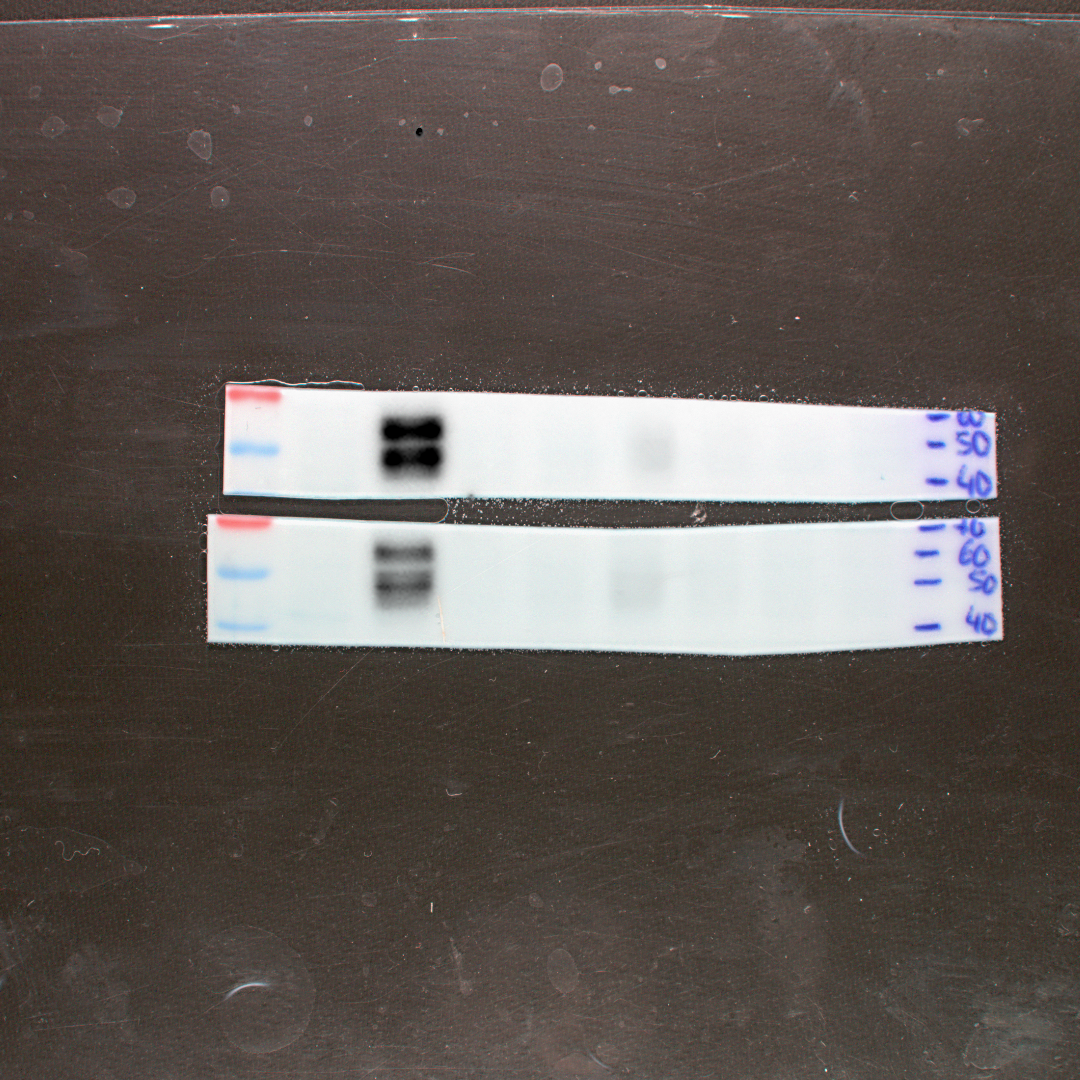

Supplement: Figure 3—figure supplement 1—source data 1. [file elife-88350-fig3-figsupp1-data1.zip › Figure 3 - figure supplement 1/DLK-1 upper membrane.Tif]

Figure 3 - figure supplement 1

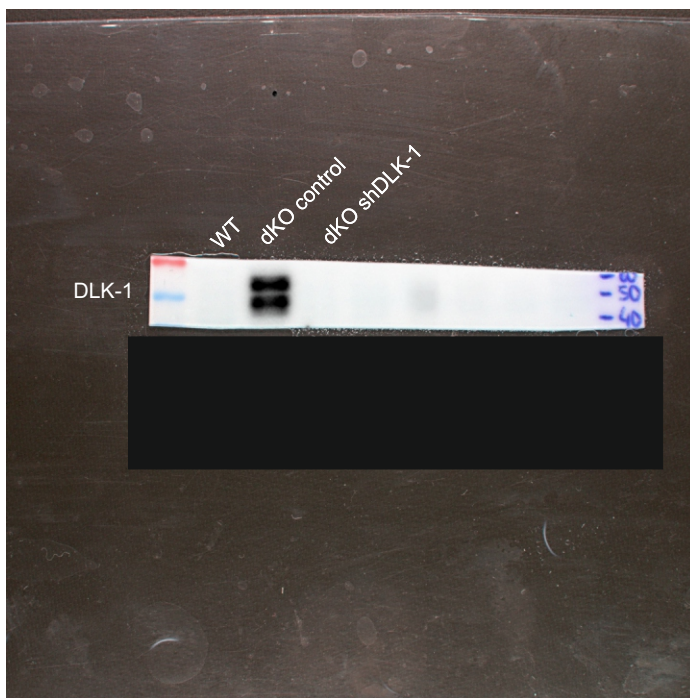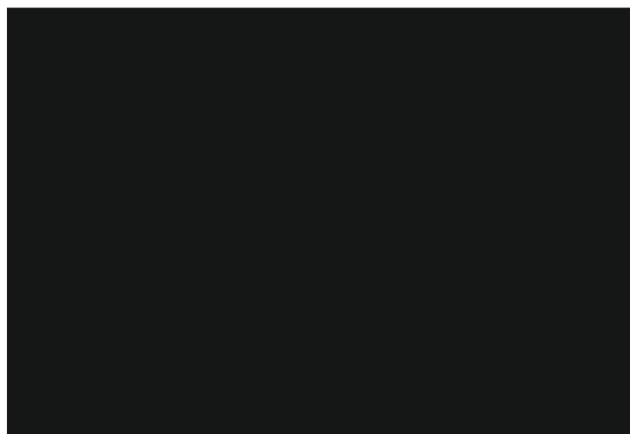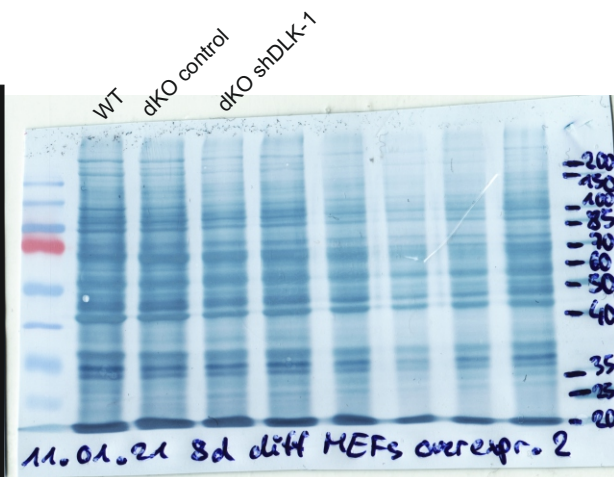

amido black

Supplement: Figure 3—figure supplement 1—source data 1. [file elife-88350-fig3-figsupp1-data1.zip › Figure 3 - figure supplement 1/Figure 3 - figure supplement 1 labeled.pdf]

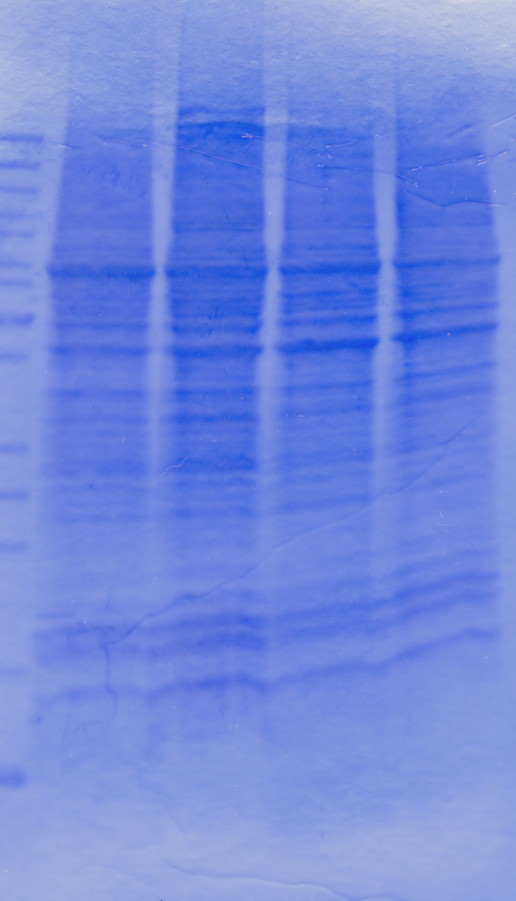

Supplement: Figure 4—source data 1. [file elife-88350-fig4-data1.zip › Figure 4A - source data/02 10 2019 Coomasie.tif]

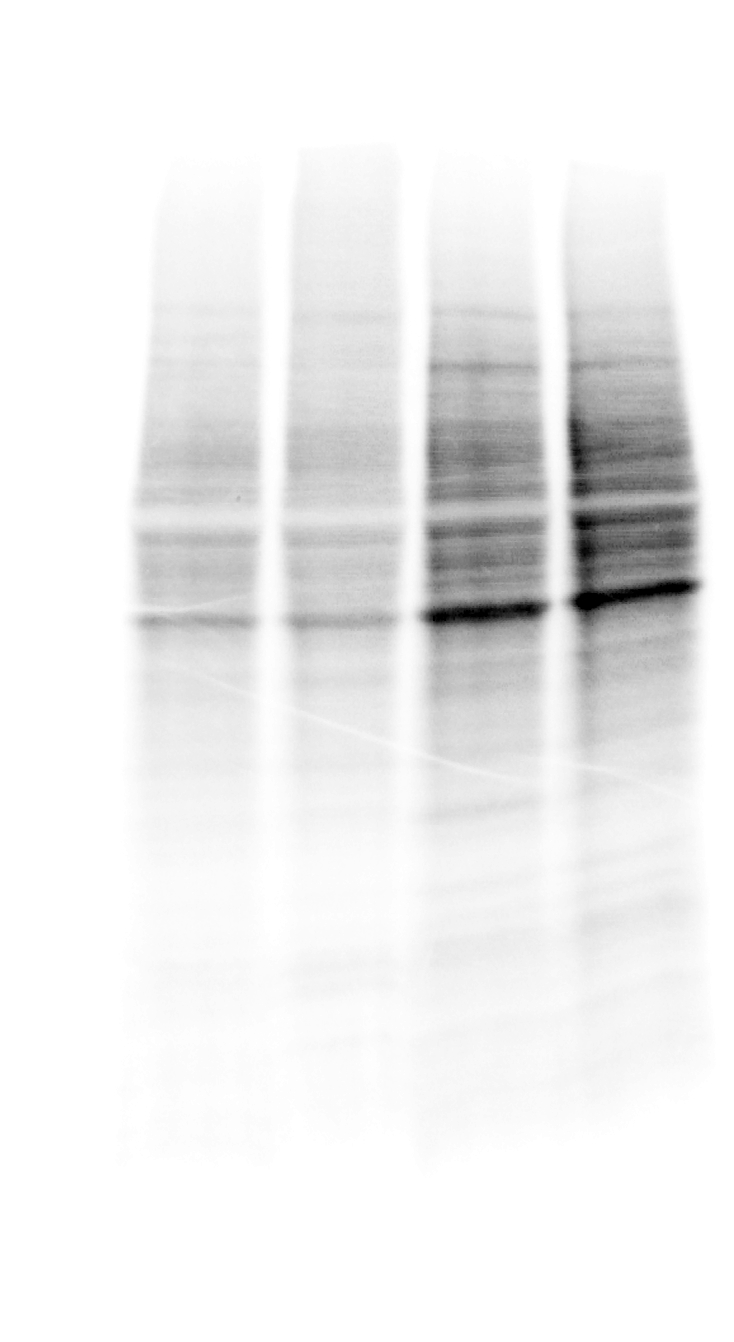

Supplement: Figure 4—source data 1. [file elife-88350-fig4-data1.zip › Figure 4A - source data/20191002-[Phosphor].jpg]

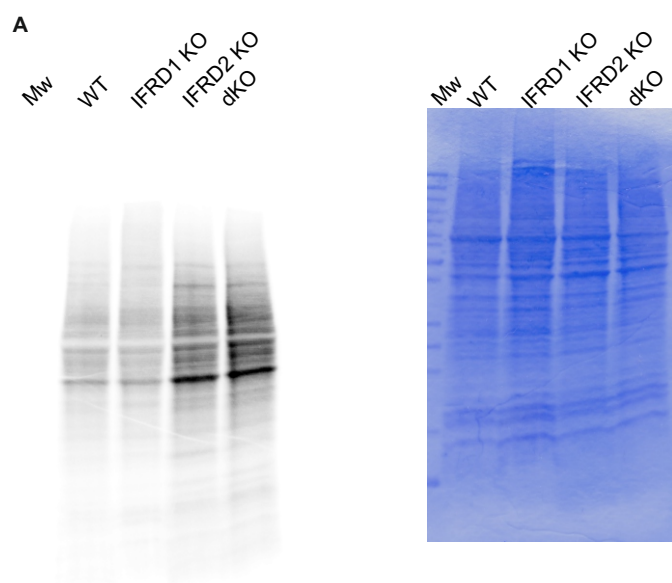

Supplement: Figure 4—source data 1. [file elife-88350-fig4-data1.zip › Figure 4A - source data/manuscript Fig 4A labeled.pdf]

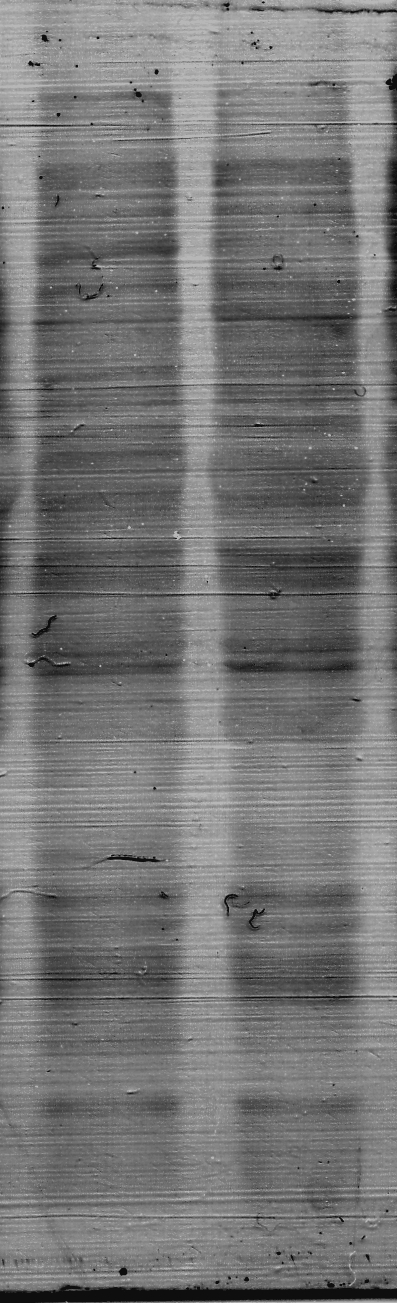

Supplement: Figure 4—source data 2. [file elife-88350-fig4-data2.zip › Figure 4B - source data/Amido black.jpg]

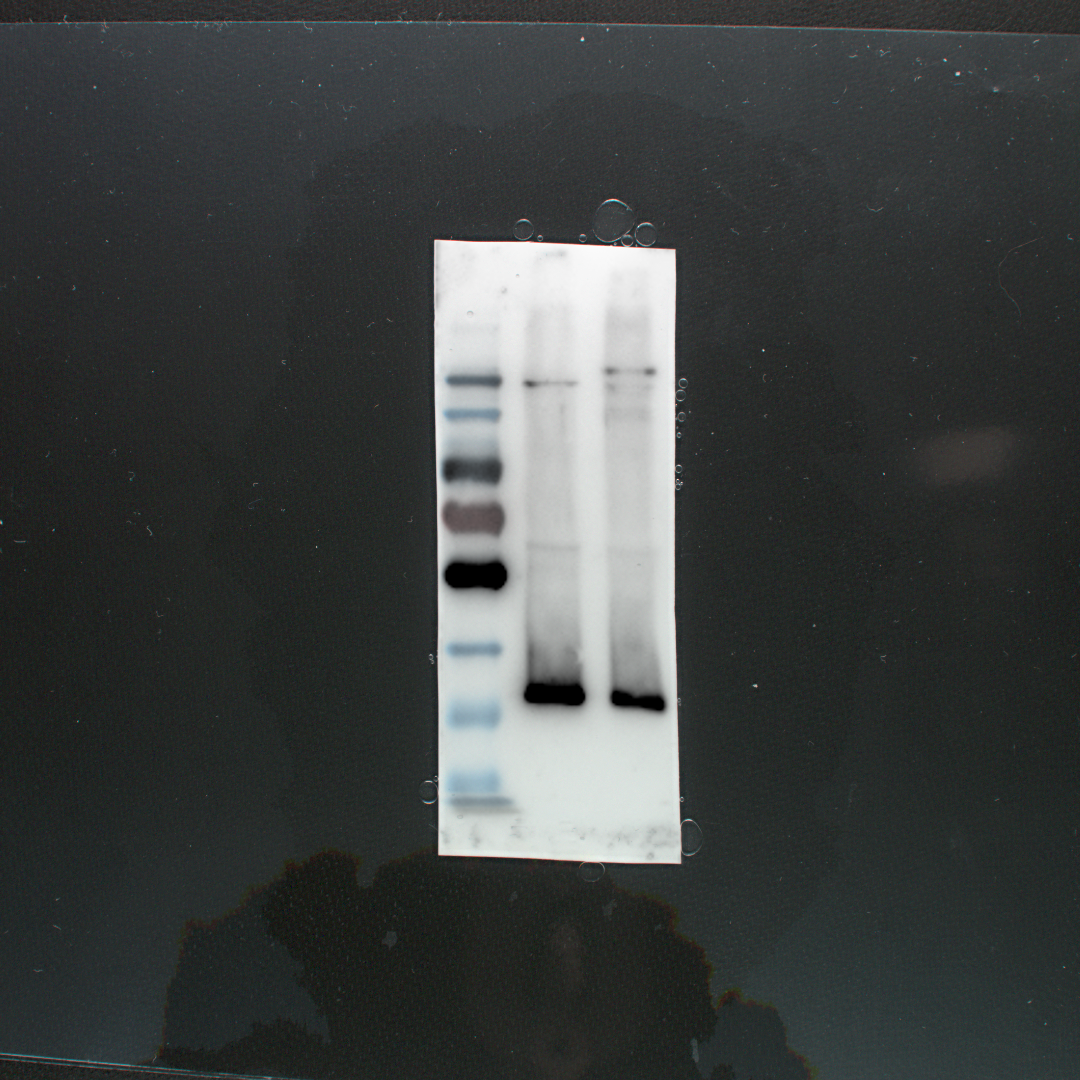

Supplement: Figure 4—source data 2. [file elife-88350-fig4-data2.zip › Figure 4B - source data/Collagen.Tif]

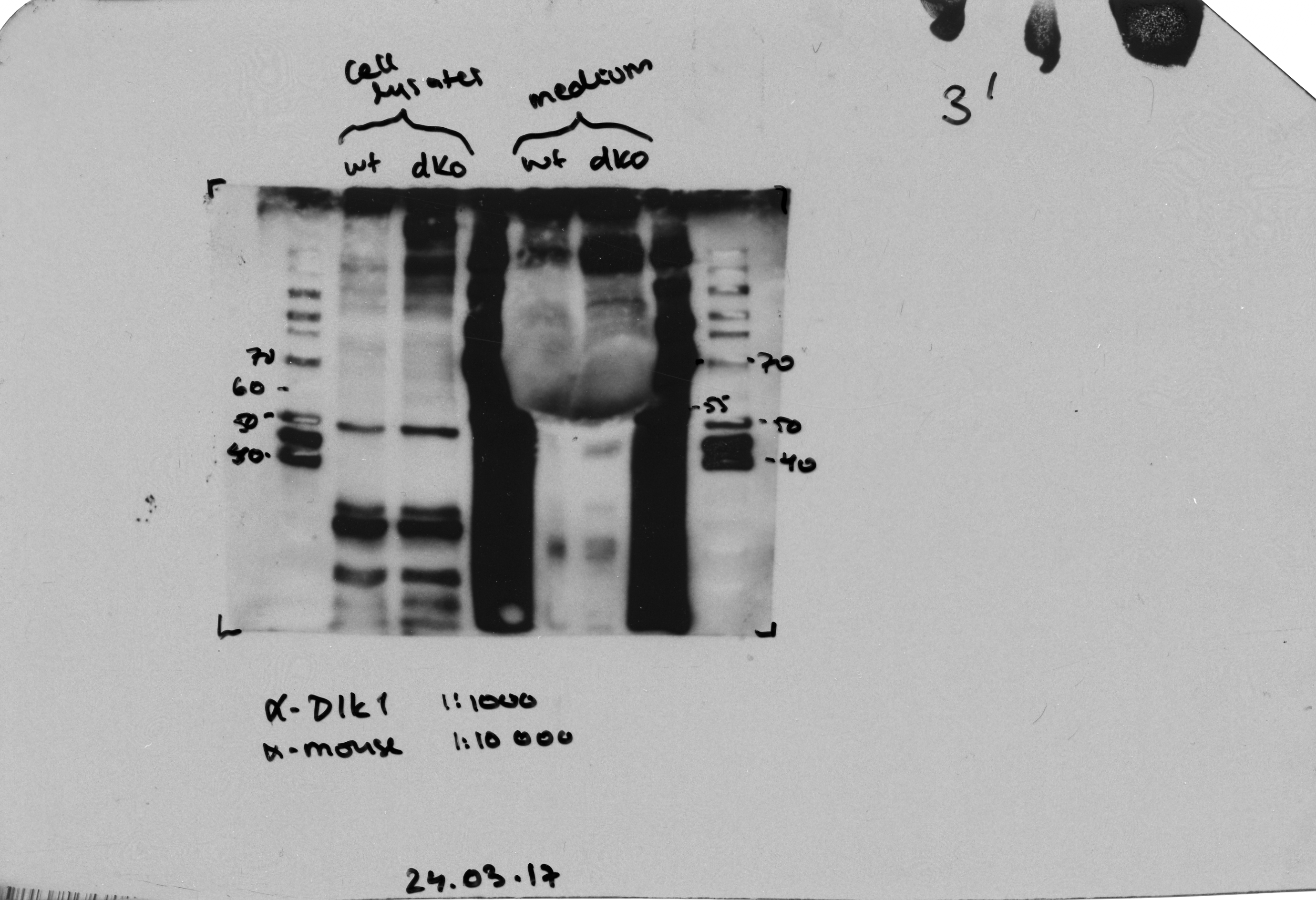

Supplement: Figure 4—source data 2. [file elife-88350-fig4-data2.zip › Figure 4B - source data/DLK1.tif]

B

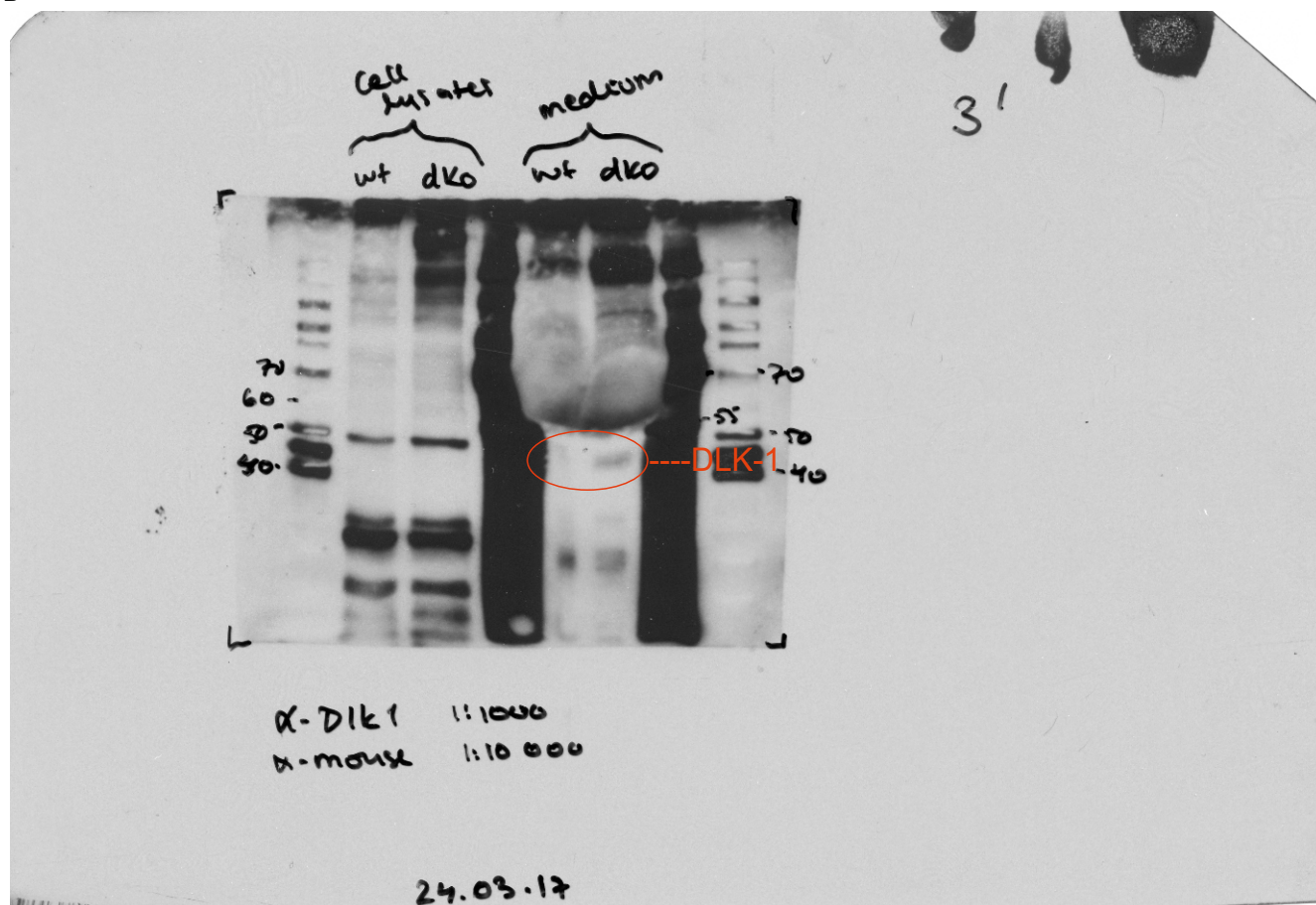

DLK-1 WB

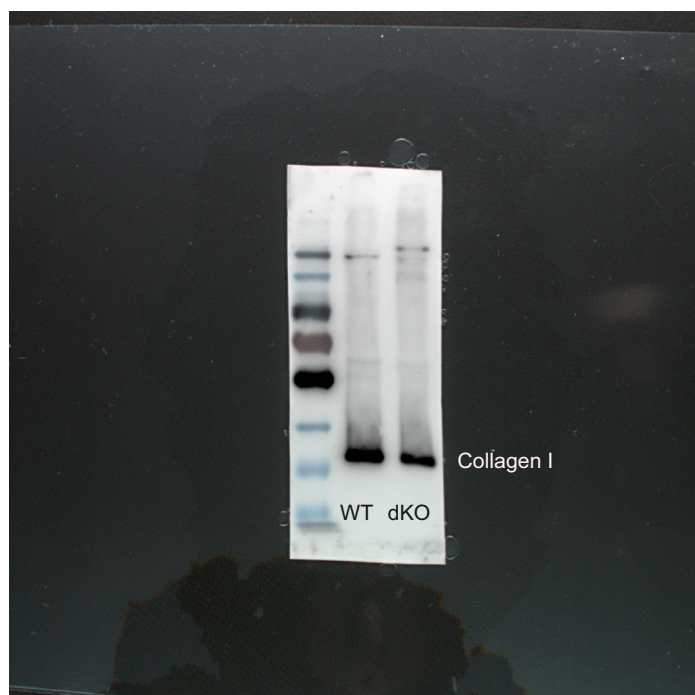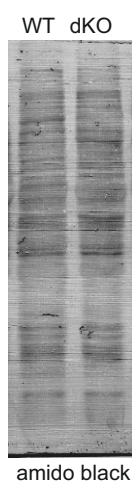

Supplement: Figure 4—source data 2. [file elife-88350-fig4-data2.zip › Figure 4B - source data/manuscript Figure 4B labeled.pdf]

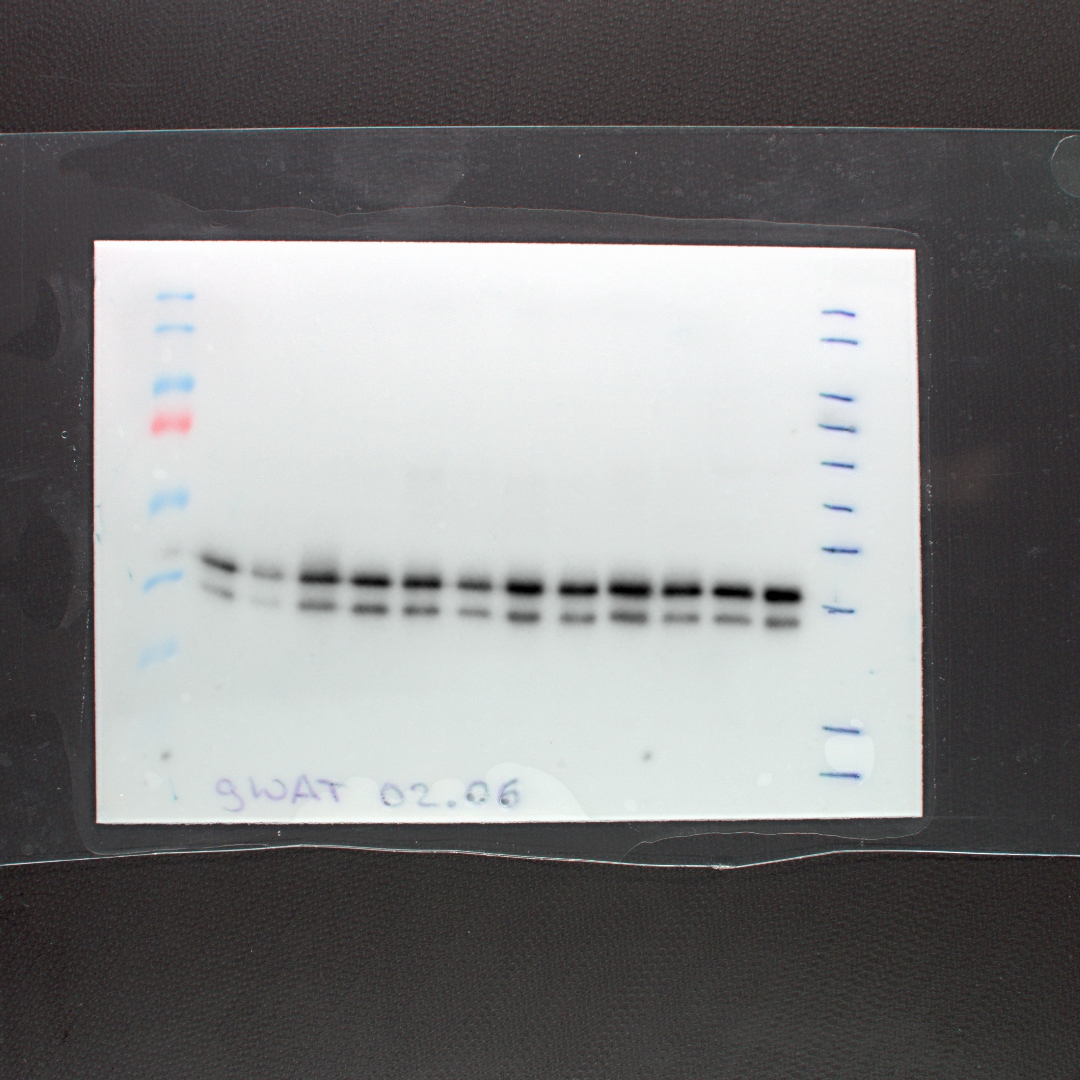

Supplement: Figure 4—source data 3. [file elife-88350-fig4-data3.zip › Figure 4G - source data/04_06_2021_p42 44 15,2s.jpg]

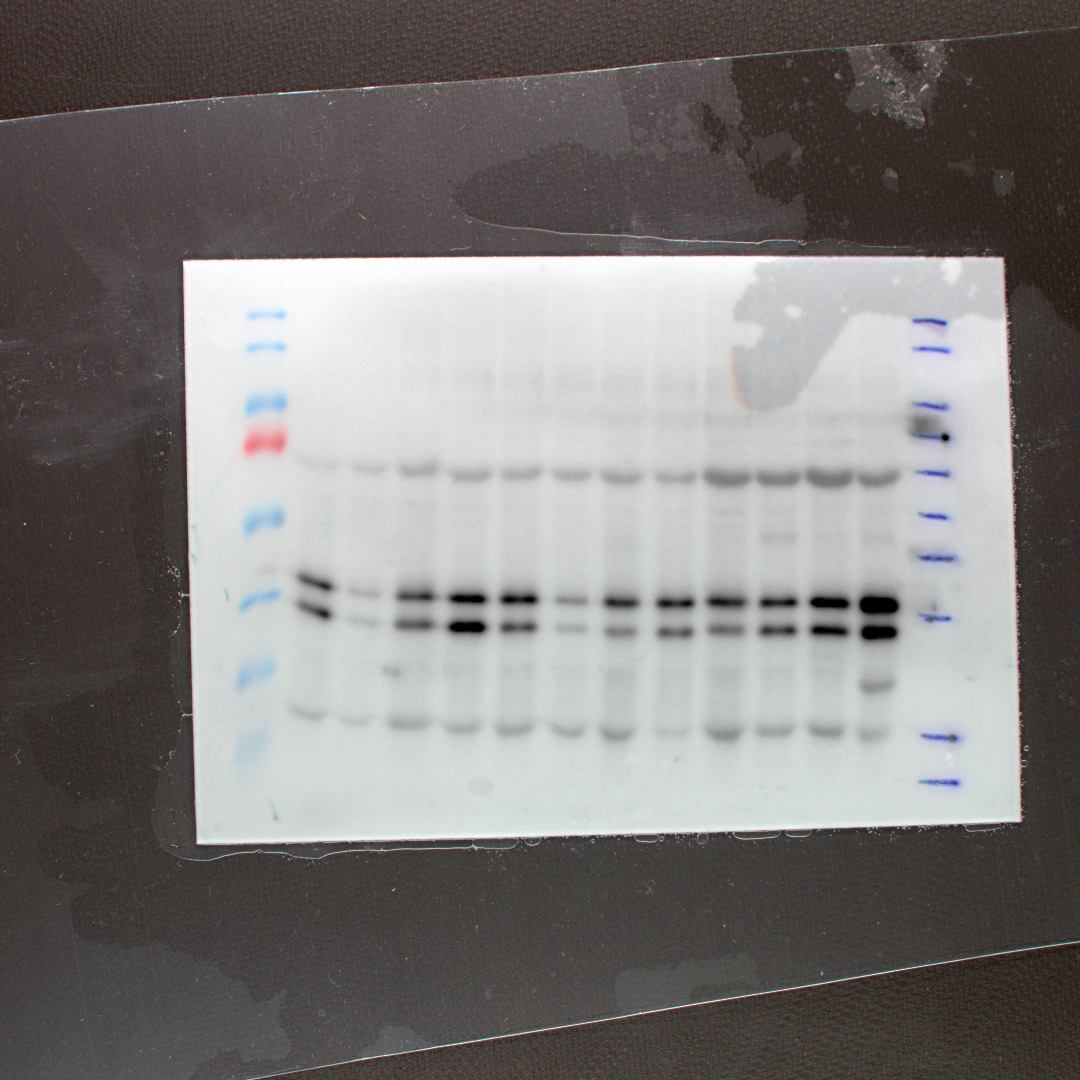

Supplement: Figure 4—source data 3. [file elife-88350-fig4-data3.zip › Figure 4G - source data/04_06_2021_Pp42 44 1min.jpg]

G

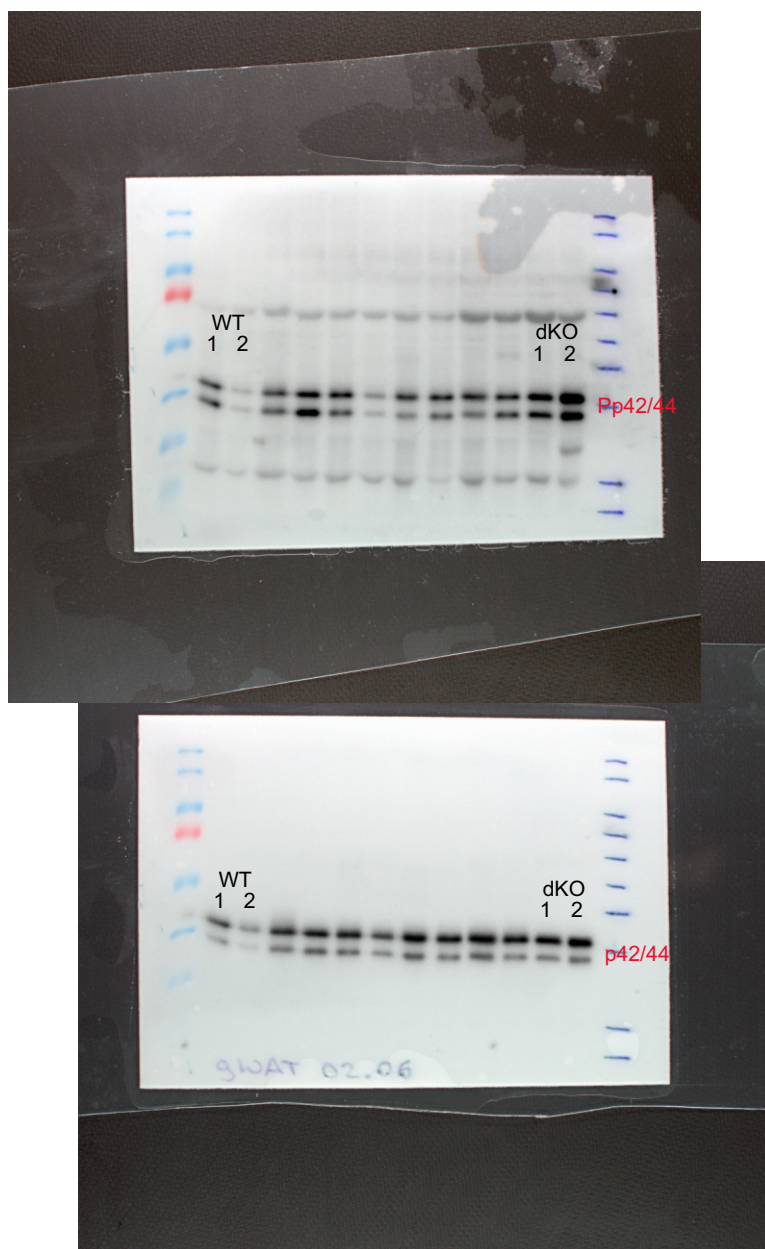

Supplement: Figure 4—source data 3. [file elife-88350-fig4-data3.zip › Figure 4G - source data/manuscript Fig 4G.pdf]

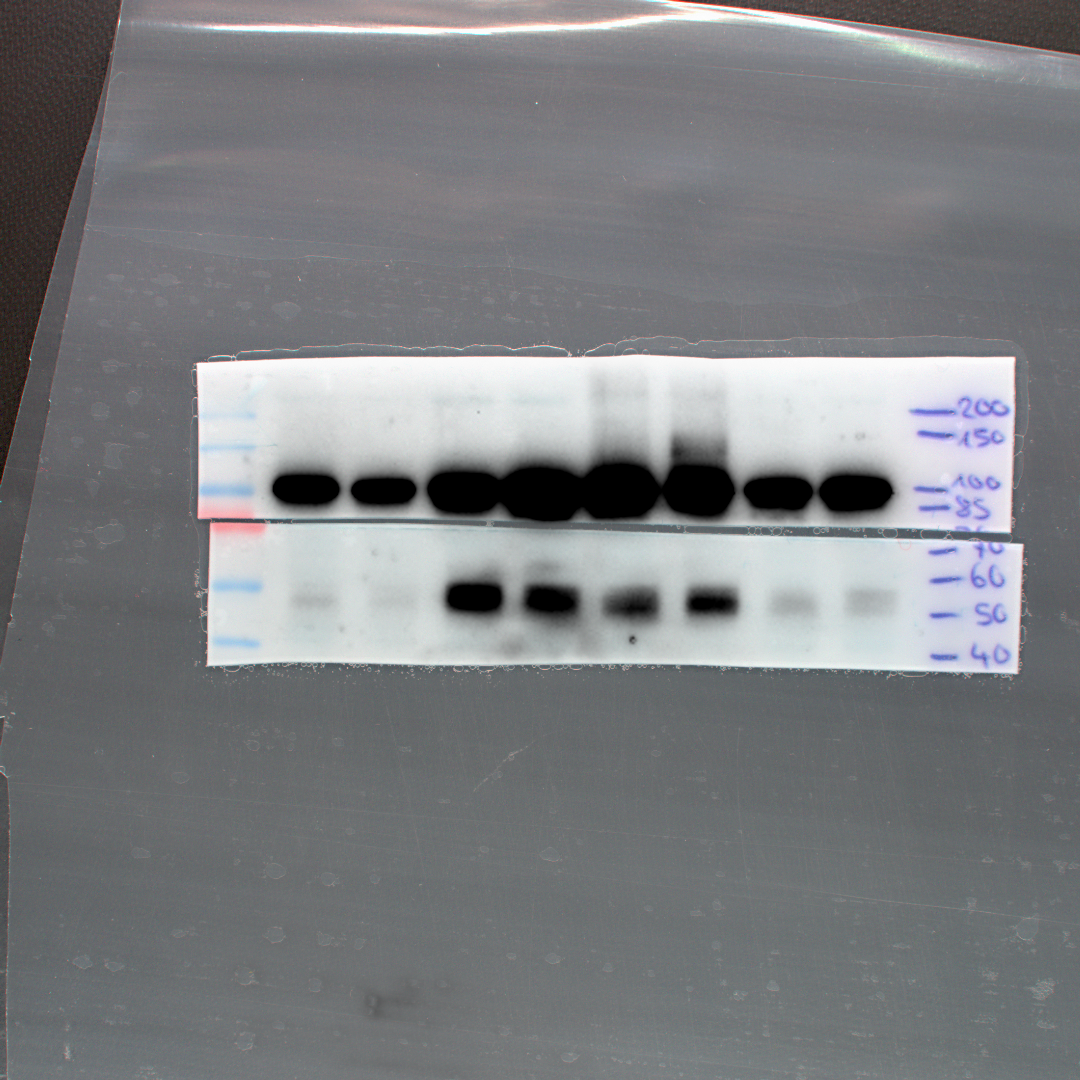

Supplement: Figure 4—source data 4. [file elife-88350-fig4-data4.zip › Figure 4I - source/DLK1 lower blot.Tif]

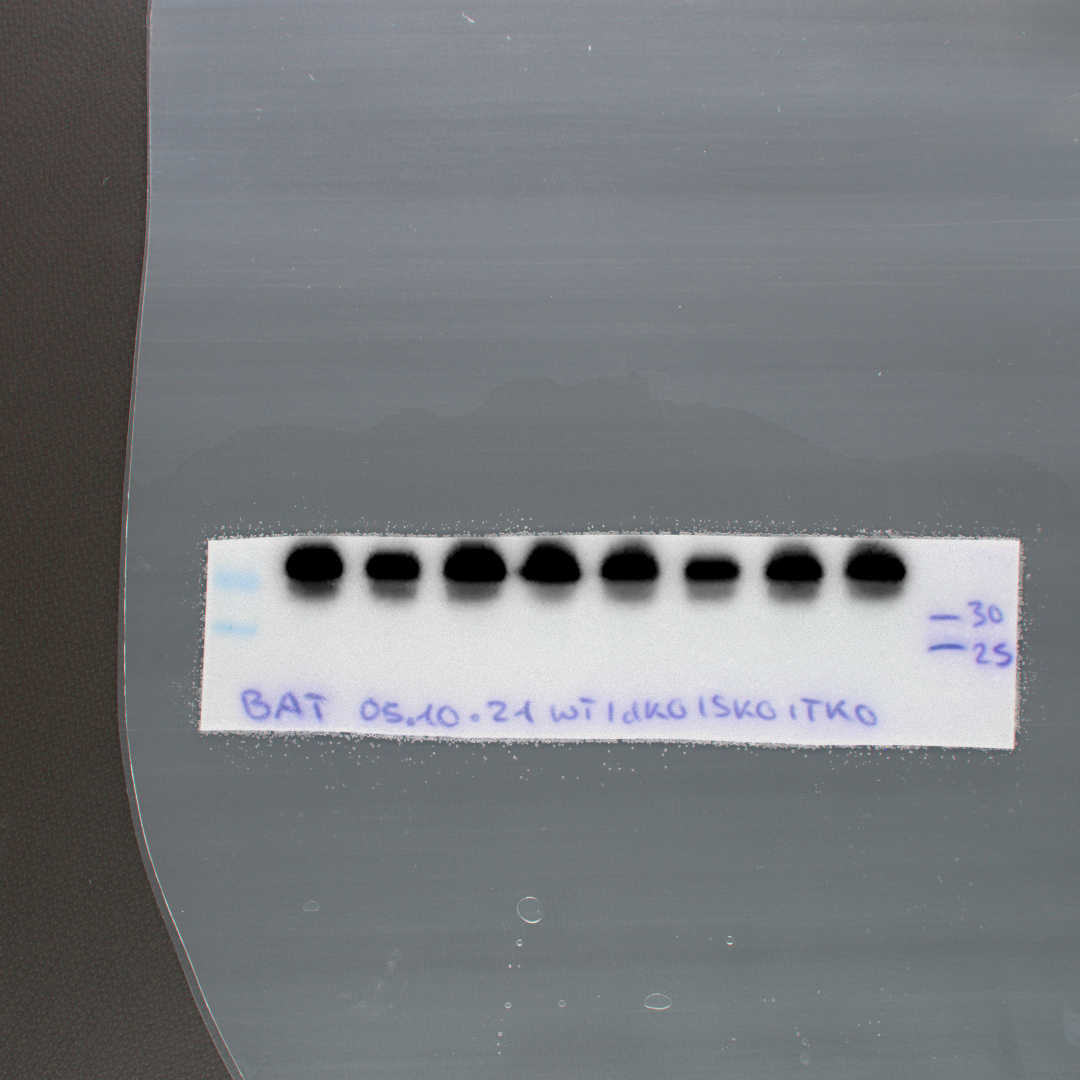

Supplement: Figure 4—source data 4. [file elife-88350-fig4-data4.zip › Figure 4I - source/GAPDH blot.Tif]

G

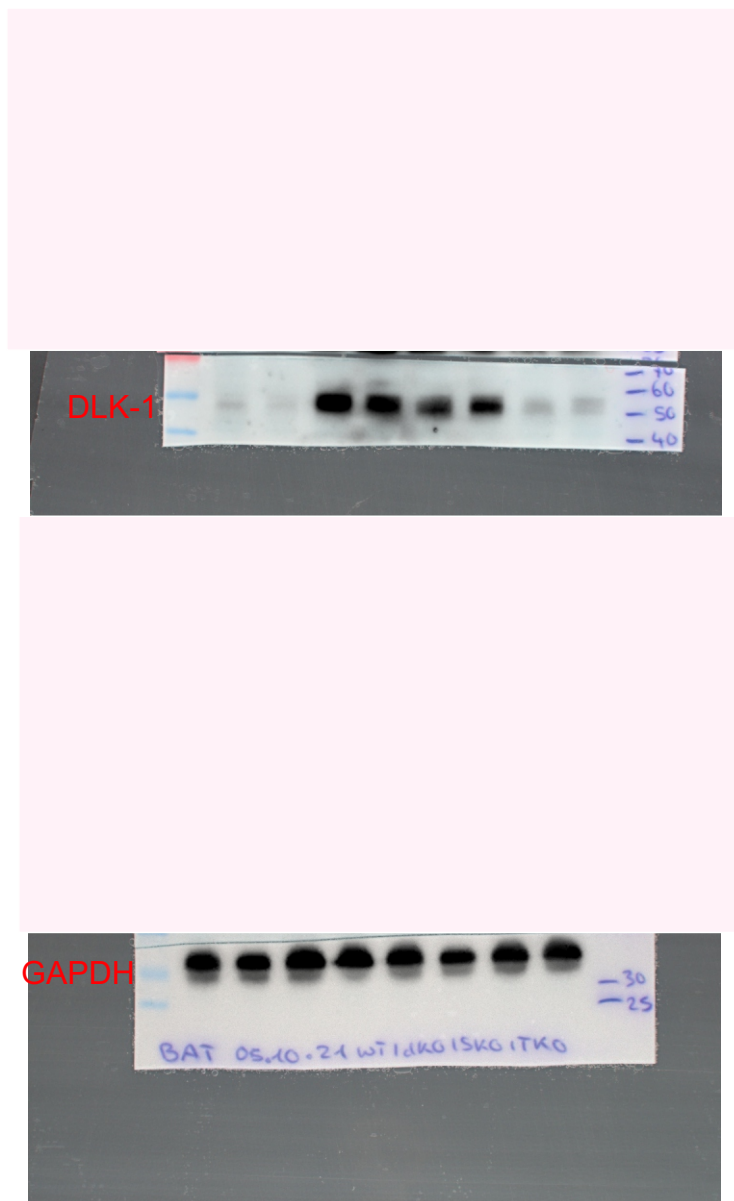

Supplement: Figure 4—source data 4. [file elife-88350-fig4-data4.zip › Figure 4I - source/manuscript Fig 4I labeled.pdf]

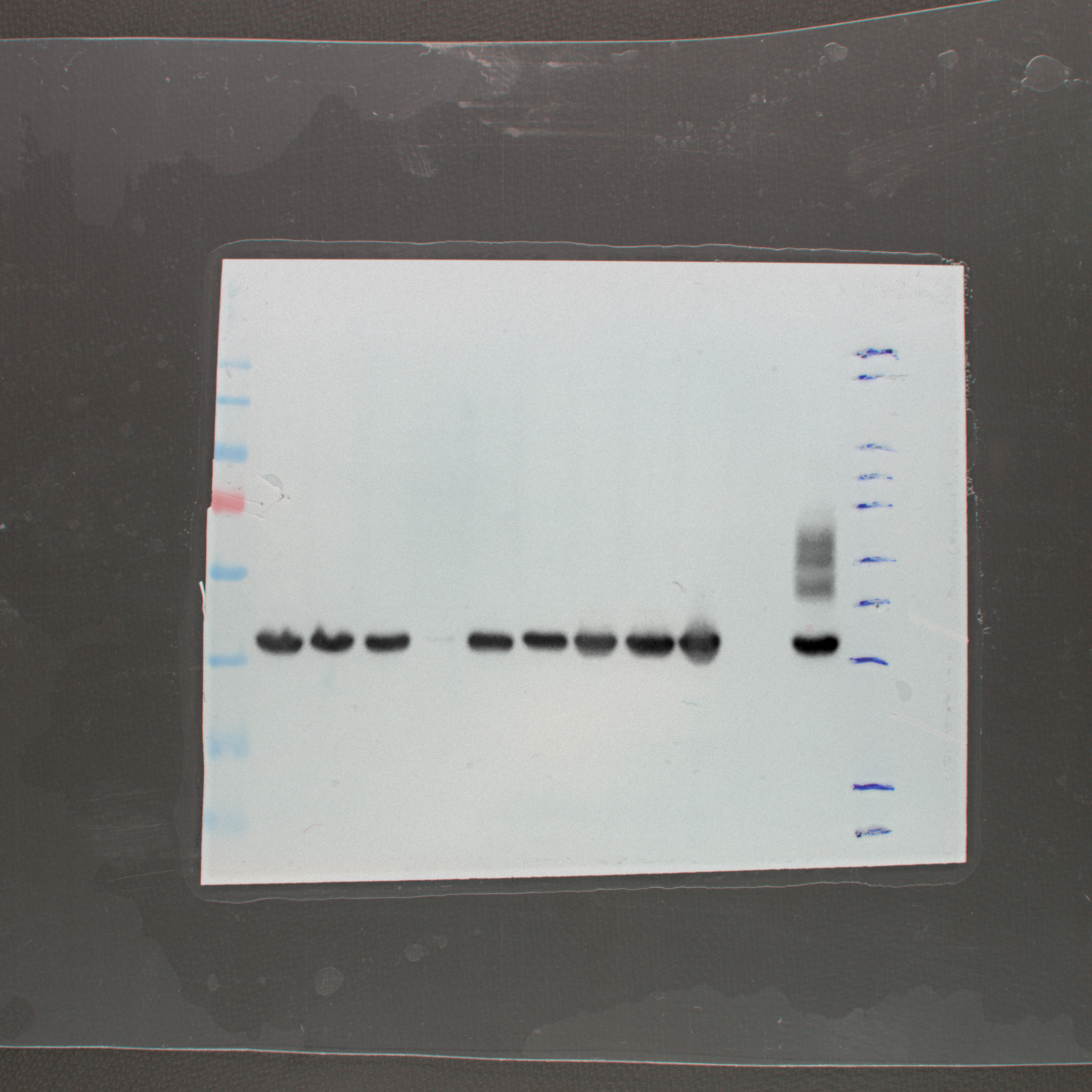

Supplement: Figure 4—figure supplement 1—source data 1. [file elife-88350-fig4-figsupp1-data1.zip › Figure 4 - figure supplements 1 and 2/18_05_2021_actin.tif]

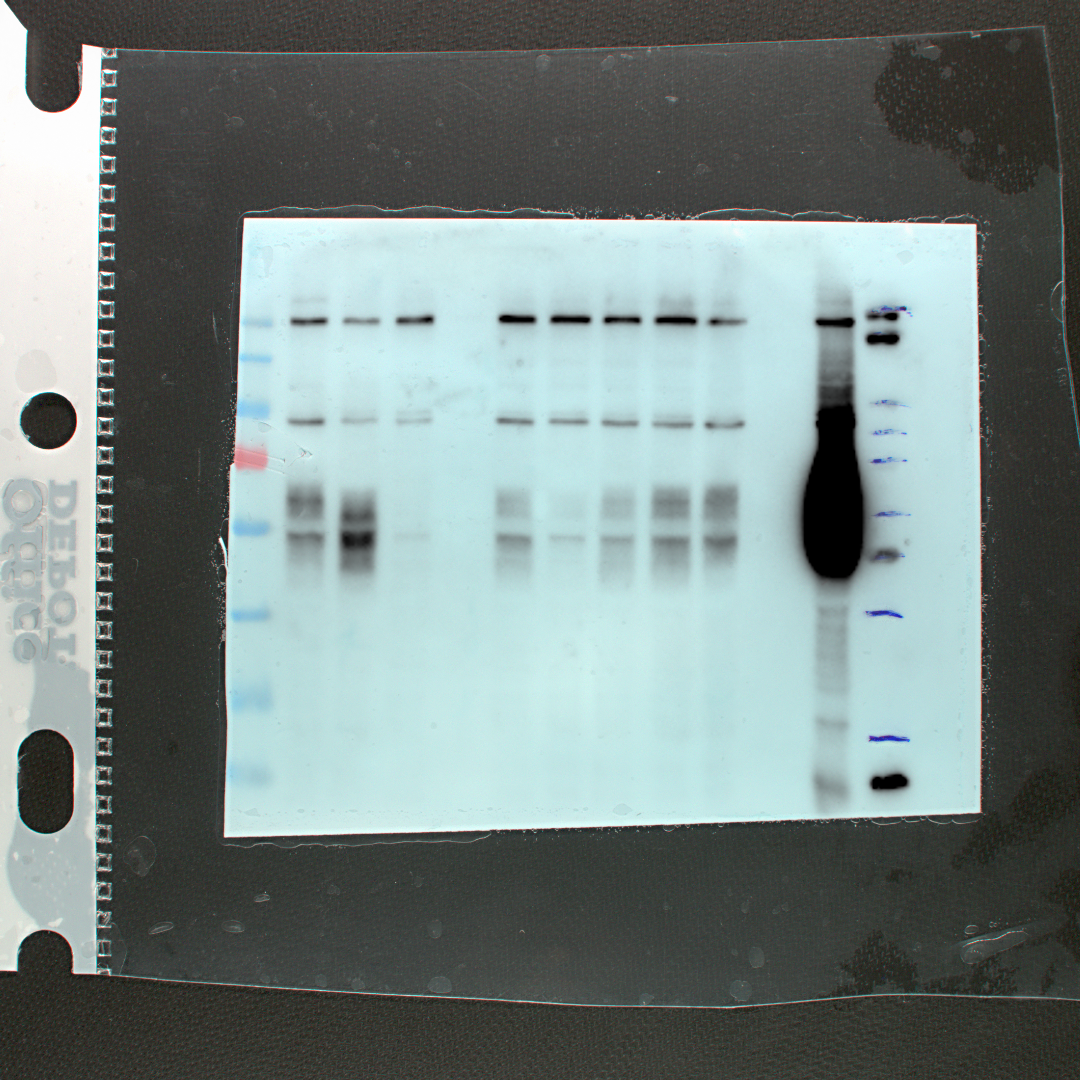

Supplement: Figure 4—figure supplement 1—source data 1. [file elife-88350-fig4-figsupp1-data1.zip › Figure 4 - figure supplements 1 and 2/18_05_2021_DLK1.tif]

Figure 4 - figure supplement 1 and 2

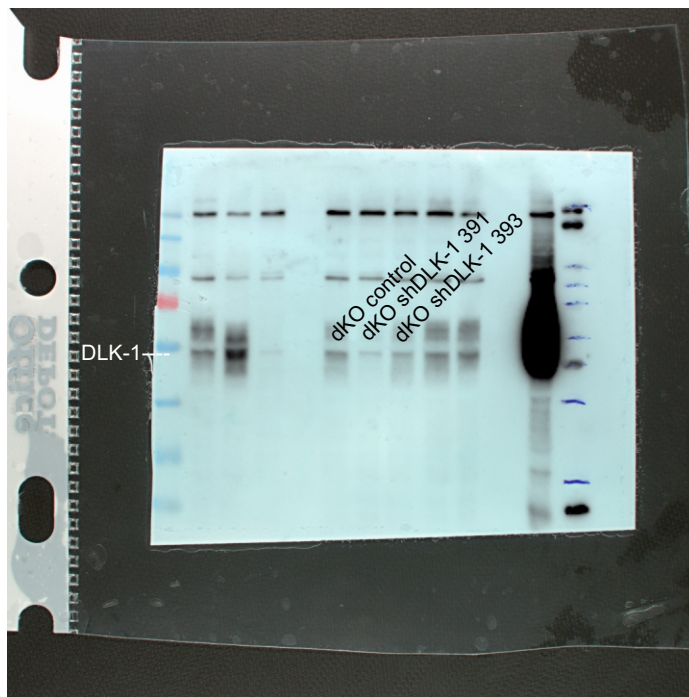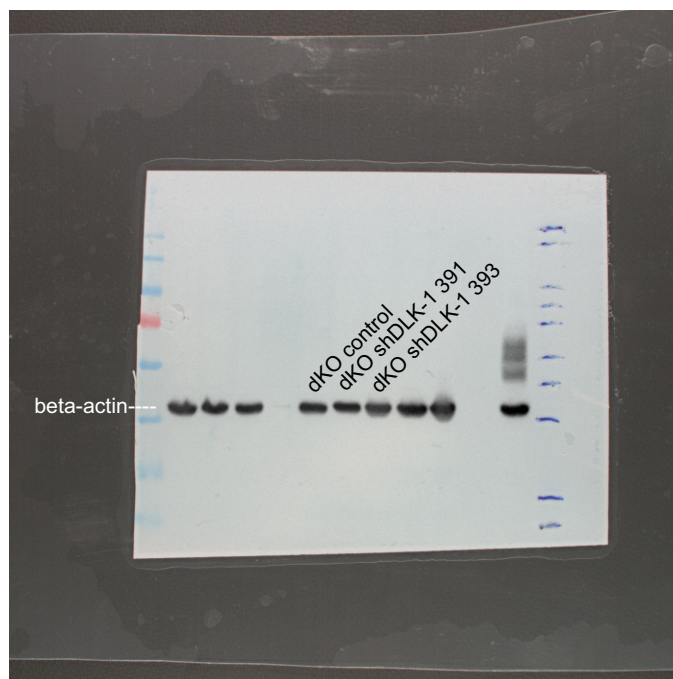

Supplement: Figure 4—figure supplement 1—source data 1. [file elife-88350-fig4-figsupp1-data1.zip › Figure 4 - figure supplements 1 and 2/manuscript Figure 4 - supplement 1 and 2 labeled.pdf]
